# HIV-1 manipulates CD96 on CD4^+^ T cells to subvert antiviral immunity

**DOI:** 10.1126/sciadv.adx7485

**Published:** 2025-09-05

**Authors:** Sandra Dehn, Rabea Burkhard, Johanna Leyens, Tabea Kaiser, Simone Brandimarte, Dinah Heiligensetzer, Herwig Koppensteiner, Baubak Bajoghli, Stephan Hailfinger, Karin Schilbach, Michael Schindler

**Affiliations:** ^1^ Institute for Medical Virology and Epidemiology of Viral Diseases, University Hospital Tübingen, Tübingen, Germany.; ^2^ Institute of Virology, Helmholtz Zentrum Munich, German Research Center for Environmental Health, Neuherberg, Germany.; ^3^ Department of Oncology, Hematology, Immunology and Rheumatology, University Hospital Tübingen, Tübingen, Germany.; ^4^ Department of Medicine A, Hematology, Oncology and Pneumology, University Hospital Münster, Münster, Germany.; ^5^ Department of Pediatric Hematology and Oncology, University Children’s Hospital Tübingen, Tübingen, Germany.

## Abstract

HIV-1 evades immune responses by modulating plasma membrane receptors. Using a flow cytometry–based screening, we profiled 332 surface receptors on HIV-1–infected primary CD4^+^ T cells and identified 23 down-regulated receptors, including known targets such as CD4, MHCI, CCR7, and CD62L. CD96, an inhibitory natural killer (NK) cell receptor poorly studied in human CD4^+^ T cells, was markedly down-regulated. This modulation, mediated by the viral proteins Nef and Vpu, surpassed that of other NK-associated receptors such as CD155 and NTB-A and is conserved across lentiviruses. CD96^Hi^ CD4^+^ T cells exhibited a proinflammatory T_H_1/T_H_17 phenotype characterized by IFN-γ and IL-17 secretion and displayed impaired migration in vivo. Furthermore, CD96 ligation enhanced IFN-γ release upon viral peptide stimulation and promoted the secretion of T_H_1/T_H_17-associated cytokines. Our findings suggest that CD96 regulates antiviral immune responses and maintains proinflammatory properties in CD4^+^ T cells. Thus, its down-regulation represents a previously unknown HIV-1 immune evasion strategy, with implications for exploiting CD96 as immunotherapeutic target.

## Teaser

HIV-1 down-regulates CD96, revealing the role for this receptor in regulating the antiviral immune responses in CD4^+^ T cells.

## INTRODUCTION

Infection and depletion of CD4^+^ T cells by HIV-1 are central to viral pathogenesis and the hallmark of AIDS progression. By infection of CD4^+^ T cells, HIV-1 infiltrates and subverts this important immune cell type necessary to orchestrate the immune response against pathogens (*1*). To efficiently persist and replicate in cells of the immune system, HIV-1 evolved sophisticated mechanisms to shut down various components of both innate and adaptive immunity (*2*, *3*). Given that immune cells communicate through direct cell-cell interactions and the secretion of cytokines, one key strategy of HIV-1 to evade immune control is to induce substantial changes in the membrane architecture. Landmark contributions in this context include the identification of HIV-1 Nef downmodulating cell surface major histocompatibility complex class I (MHCI) to prevent the killing of infected T cells by cytotoxic T lymphocytes and the discovery of CD4 downmodulation by the concerted action of HIV-1 Nef, Vpu, and Env (*4*–*6*). More recently, Vpu was found to interfere with the interferon-induced antiviral factor Tetherin (*7*, *8*) and receptors necessary for natural killer (NK) cell responses (*9*–*12*). Both Nef and Vpu are so-called HIV-1 accessory viral proteins; while dispensable for virus replication in various immortalized cell lines, these proteins are critical for achieving high viral titers in vivo. Thus, Nef and Vpu contribute to the pathogenicity of HIV-1, CD4^+^ T cell depletion, and progression to AIDS (*13*, *14*). Hence, a thorough understanding of HIV-1–induced perturbation at the plasma membrane will allow a better understanding of viral immunopathogenesis and enhance our knowledge of immune cell functioning and signaling.

Previous studies aiming to broadly assess HIV-1–induced membrane remodeling either used transfected T cell lines overexpressing HIV-1 Nef and Vpu (*15*) or performed comprehensive membrane proteomics in the context of infection, also using an immortalized T cell line (*16*). Our group conducted similar membrane profiling on HIV-1–infected macrophages, where we found minimal virus-induced changes in surface receptor expression (*17*). These findings underscore the importance of studying specific primary cell types, as the extent of HIV-1–mediated modulation may vary substantially between different immune cells. In consequence, up to now, there is no study analyzing HIV-1–induced alterations of the cell membrane on a broader scale in infected primary CD4^+^ T cells. Accordingly, the objective of this study was to investigate plasma membrane perturbations in productively HIV-1–infected primary CD4^+^ T cells. We established an unbiased, phenotypic flow cytometry–based screen using an arrayed panel of 332 antibodies to identify receptors on the surface of primary CD4^+^ T cells, which are specifically modulated upon HIV-1 infection. The screening method was validated by identifying receptors already known to be modulated by HIV-1. We found several previously unknown or poorly described surface receptors affected by HIV-1. Among them, the putative NK cell receptor CD96.

CD96, also known as TACTILE (T cell activation, increased late expression), is a type 1 transmembrane glycoprotein belonging to the immunoglobulin (Ig) superfamily (*18*). Primarily expressed on T cells and NK cells, CD96 binds to its ligand PVR (poliovirus receptor; CD155) (*19*). Humans, unlike mice, express two distinct splice variants of CD96 that differ in their second Ig domain. The variant 2 (CD96v2) is predominant and considered as the canonical form (*20*). Structurally, CD96 consists of three extracellular IgG-like domains, of which the outermost V-like domain is responsible for ligand binding and a C-like domain that anchors the receptor to the plasma membrane via the stalk region (*21*, *22*). The intracellular part of CD96 contains multiple conserved signaling motifs with unclear functionality. A CX_8_RK motif may facilitate the association of SRC-like kinases, while proline-rich tandem motifs potentially serve as Src homology 3–binding sites. Moreover, the intracellular CD96 region contains a putative immunoreceptor tyrosine-based inhibitory motif (ITIM) and an additional putative activating YXXM motif, which is present in human CD96 (hCD96) but absent in its murine ortholog (*21*–*23*). Conceivably, because of the numerous possible functions attributed to the various signaling motifs, predicting the functional role of CD96 based on structure-motif associations is challenging.

CD96 is part of an NK cell signaling receptor network. CD155 (PVR), expressed as a membrane-bound protein on the surfaces of dendritic cells, macrophages, and tumor cells, not only interacts with CD96 but also binds other Ig superfamily receptors, such as TIGIT (T cell immunoreceptor with Ig and ITIM domains) and CD226 (platelet and T cell activation antigen 1; DNAM-1), which share sequence homology in the first V domain of the extracellular region. This CD96/TIGIT/CD226 axis forms a complex signaling network that both stimulates and inhibits immune cell function and cytokine production upon binding to CD155 (*23*). While TIGIT leads to a decrease in NK cell activity (*24*, *25*), CD226 promotes activation, proliferation, and differentiation of NK cells (*26*, *27*). The role of CD96 within this network, however, remains controversial, as its function could be dependent on several factors such as cell type, ligand binding, and organism. The situation is further complicated by conflicting studies reporting enhancing effects of hCD96 in the context of NK cell–mediated cytotoxicity (*19*) versus a suppressing role of murine CD96 (mCD96) on NK cell activity (*28*). The same holds true for CD8^+^ T cells, where both hCD96 and mCD96 have been described as costimulatory (*29*) and immunosuppressive (*30*). However, as NK cell inhibitory immune checkpoint receptor, CD96 is considered a potential immunotherapeutic target (*22*, *31*, *32*). A combined treatment involving anti–PD-1 monoclonal antibody (mAb), anti–CTLA-4 mAb, and anti-CD96 mAb reduced metastasis and prolonged survival in murine models (*33*). However, because of the conflicting data obtained from studies in mice and humans regarding whether hCD96 activates or inhibits NK cell function, further translational development into human therapy is slowed down. Overall, while the role of CD96 on NK cells and CD8^+^ T cells is subject to intensive investigations, its function on CD4^+^ T cells remains poorly understood. Therefore, the modulation of CD96 in productively HIV-1–infected CD4^+^ T cells may provide critical insights into its role in human immunobiology and lever our understanding of its role in T cell signaling and regulation, especially in the context of infection and inflammation.

Together, we here present a quantitative unbiased expression analysis of 332 membrane receptors in primary CD4^+^ T cells and their modulation as a consequence of HIV-1 infection. By this, our work generates a blueprint to understand the mechanisms of HIV-1–mediated immune evasion in CD4^+^ T cells. Furthermore, we find CD96 as immune receptor being down-regulated by HIV-1 and propose a functional role for CD96-expressing human CD4^+^ T cells.

## RESULTS

### The plasma membrane receptor expression pattern of HIV-1–infected CD4^+^ T cells

**Fig. 1.**
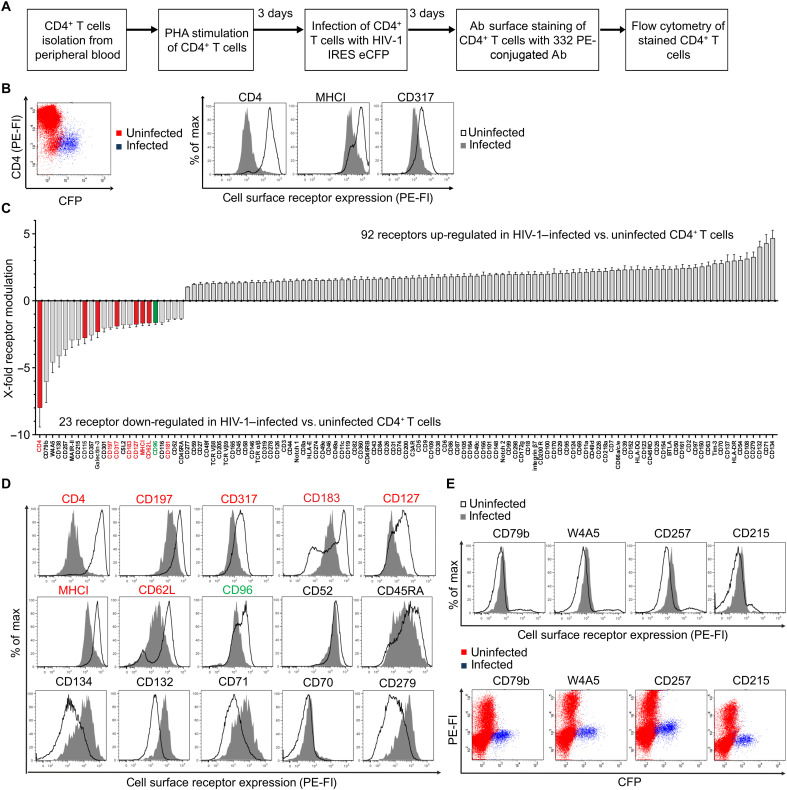
Screening for cell surface receptor modulations in HIV-1–infected primary CD4^+^ T cells. (**A**) Scheme of the experimental setup of the surface screen. Analysis used PE MFI values from both infected (eCFP^+^ cells) and uninfected cells (eCFP^−^ cells). Ab, antibody. (**B**) Representative dot plot and histograms from analysis of one donor showing the down-regulation of CD4, CD317 (Tetherin), and MHCI (HLA-ABC). FI, fluorescence intensity. (**C**) Water fall plot displaying fold receptor modulations at the plasma membrane of productively HIV-1–infected CD4^+^ T cells directly related to uninfected cells. Selected down-regulated genes that were confirmed by this screen are labeled in red, and CD96 down-regulation is shown in green. The *P* value for all receptors shown is <0.05 calculated with a paired *t* test from the 13 donors sampled here and corrected for multiple hypothesis testing. (**D**) Representative histograms of selected receptor modulations in HIV-1–infected versus uninfected CD4^+^ T cells. (**E**) Representative histograms and dot plots of cell surface receptors that identify CD4^+^ T cells, which are not permissive for productive HIV-1 infection. The underlying raw data are included in dataset S1.

To profile HIV-1–induced plasma membrane perturbations, we established a flow cytometry–based screen of ex vivo infected primary CD4^+^ T cells (Fig. 1A). Using an enhanced cyan fluorescent protein (eCFP)–expressing full-length HIV-1 reporter construct, we selectively identified the infected cell population based on eCFP expression, enabling screening without compensating for fluorescence artifacts due to minimal spectral overlap between eCFP and the phycoerythrin (PE)–conjugated antibody fluorescence (Fig. 1B, left). For visualization of potential receptor modulations, PE mean fluorescence intensity (MFI) values of uninfected (eCFP^−^) and productively infected (eCFP^+^) CD4^+^ T cells populations in the same culture are shown as histograms (Fig. 1B). In line with expectations and thus validating our screening approach, we observe clear HIV-1–induced down-regulation of CD4, CD317 (Tetherin), and MHCI, as shown in the representative donor (*5*, *6*, *8*).

To account for donor variations, we performed the screen with primary CD4^+^ T cells from 13 different donors. Data analysis and filtering were essentially done as before (*17*, *34*). The primary data were prescreened for potential technical issues during the staining procedure and acquisition, with any affected single data points manually excluded from further analysis (dataset S1, red filled values). Then, we determined the background MFI according to the blank values and the 10 isotype controls included in the screen and marked all values below the threshold in red (dataset S1, values in red). For quantification, MFI values of uninfected cells (eCFP^−^) and infected (eCFP^+^) cell populations were compared using a two-tailed paired *t* test across multiple donors (dataset S1). For receptors showing significant modulation, we calculated the X-fold HIV-1–mediated up- or down-regulation by dividing the MFI values of infected through uninfected populations (receptors up) or vice versa (receptors down). Mean values and the corresponding SEM are depicted in a waterfall plot representing HIV-1–induced cell surface receptor modulations on productively infected CD4^+^ T cells relative to uninfected cells (Fig. 1C).

In our assay, HIV-1 induced the expression of 92 receptors, whereas 23 of the analyzed receptors were down-regulated. Receptors up-regulated by HIV-1 were largely associated with T cell activation, cell adhesion, and regulation thereof, in line with the ability of HIV-1 to cause generalized immune activation (fig. S1). To the contrary, down-regulation of certain cell surface receptors may interfere with immune control of productively infected cells (fig. S1). Notably, confirming the reliability of our screen, we identify several receptors known to be actively downmodulated by HIV-1 (Fig. 1, C and D, marked in red), including CD4 (*5*), CD317 (Tetherin) (*8*), the chemokine receptors CCR7 (CD197) (*35*), CXCR3 (CD183), and CXCR1 (CD181) (*36*), and interleukin-7 receptor (IL-7R) (CD127) (*37*). Most of the latter are known to be modulated by the action of viral accessory proteins Nef and Vpu, both being also responsible for down-regulation of human lymphocyte antigen (HLA) family receptors (MHCI) (*6*, *38*) and CD62L (*39*). One receptor that caught our attention, previously unreported to be down-regulated in HIV-1–infected primary CD4^+^ T cells, was the putative NK cell receptor CD96 (Fig. 1, C and D, labeled in green), also called TACTILE.

Notably, four receptors—CD79b, W4A5, CD257, and CD215—were observed only in a subpopulation of CD4^+^ T cells. Primary flow cytometry data revealed that productive HIV-1 infection, as indicated by eCFP expression, was exclusive to cells lacking these aforementioned receptors (Fig. 1E). Hence, it is tempting to speculate that a specific receptor cell surface expression pattern might be predictive for low permissiveness toward HIV-1 infection.

Together, our unbiased flow cytometry–based medium-throughput screen of productively HIV-1–infected primary CD4^+^ T cells identifies virus-induced perturbations at the plasma membrane, including the previously unreported down-regulation of CD96. These modulations may represent host cell responses to virus infection, biomarkers indicating high or low permissiveness toward infection, or virus-induced changes that enhance replication or facilitate immune evasion.

### HIV-1 down-regulates CD96 by the concerted action of Vpu and Nef

**Fig. 2.**
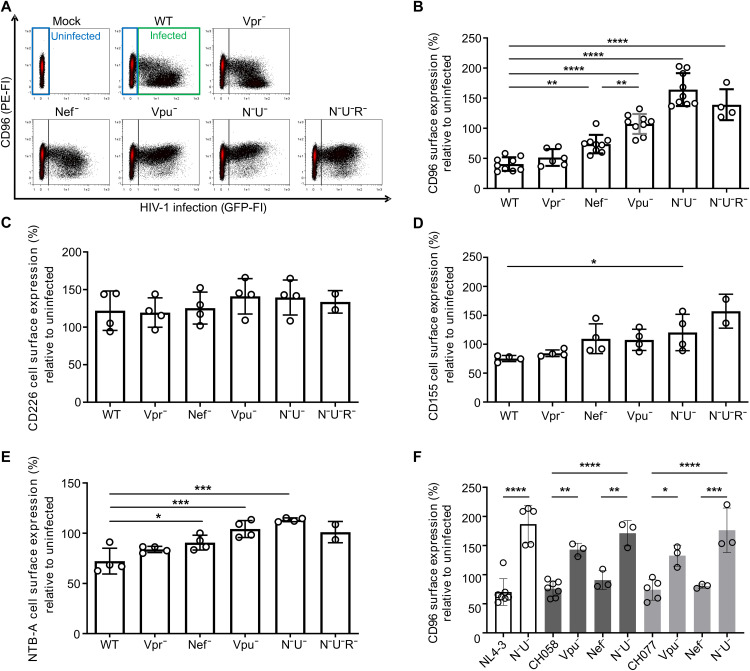
Downmodulation of cell surface CD96 from HIV-1–infected primary CD4^+^ T cells by Nef and Vpu. Primary CD4^+^ T cells were infected with HIV-1 NL4-3–IRES–eGFP reporter virus containing the indicated deletions in *vpr*, *nef*, and *vpu*. At 48 hours postinfection, cells were harvested and stained for the indicated cell surface receptors with specific antibodies and analyzed by flow cytometry. (**A**) Representative dot plots of HIV-1–infected primary CD4^+^ T cells that were stained for CD96 cell surface expression. GFP was used to identify HIV-1–infected cells (green box), and modulation was directly compared to uninfected cells (blue box). (**B**) CD96, (**C**) CD226, (**D**) CD155, and (**E**) NTB-A MFIs were used to calculate the respective surface expression of HIV-1–infected cells compared to uninfected cells. (**F**) Infection of primary CD4^+^ T cells with HIV-1 NL4-3 and two HIV-1 strains isolated from PLWH during the chronic stage (CH058 and CH077) harboring inactivating mutations in *vpu* and *nef* and assessment of cell surface CD96 levels via flow cytometry at 3 days postinfection. Values from (B) nine (NL4-3, Nef^−^, Vpu^−^, and N^−^U^−^), six (Vpr^−^), and four (N^−^U^−^R^−^) and for [(C) to (E)] four and two (N^−^U^−^R^−^) independent experiments were plotted (means ± SD). (F) Data from three to five independent donors (means ± SD), statistical differences were assessed using a one-way analysis of variance (ANOVA) with Dunnett’s multiple comparison test. ****P < 0.0001, ***P < 0.001, **P < 0.01, and *P < 0.05.

Next, we focused on CD96, a putative NK cell receptor expressed on CD4^+^ T cells, as its modulation might represent a novel mechanism through which HIV-1 evades NK cell–mediated killing. We first sought to analyze whether CD96 is down-regulated by any of the accessory HIV-1 proteins Vpr, Nef, or Vpu. For this, primary CD4^+^ T cells were infected with HIV-1 NL4-3–internal ribosomal entry site (IRES)–enhanced green fluorescent protein (eGFP) reporter viruses containing individual or combined inactivating mutations in *vpr*, *nef*, and *vpu* [NL4-3 wild-type (WT), Vpr^−^, Nef^−^, Vpu^−^, Nef^−^ Vpu^−^ (N^−^U^−^), and Nef^−^ Vpu^−^ Vpr^−^ (N^−^U^−^R^−^)]. At 48 hours postinfection, CD4^+^ T cells were harvested, stained for cell surface CD96 using anti-CD96–PE–specific antibody, and analyzed via flow cytometry (Figs. 2, A and B). HIV-1–infected cells were identified by their eGFP expression, and CD96 cell surface expression is depicted on the *y* axis. This analysis demonstrated robust and pronounced down-regulation of cell surface CD96 in HIV-1 WT–infected CD4^+^ T cells. The down-regulation was not affected by Vpr but was less pronounced when either Nef or Vpu was inactivated and fully abolished upon combined inactivating mutations in *nef* and *vpu* (N^−^U^−^; Fig. 2A). For quantification, the MFI of uninfected bystander cells (blue box) was divided by the one of productively HIV-infected cells (Fig. 2A, green box), showing that HIV-1 significantly down-regulates CD96 from the cell surface and that this phenotype is dependent on functional Nef and Vpu expression (Fig. 2B). Furthermore, Vpu was found to be more efficient at down-regulation of CD96 than Nef.

To compare the extent of CD96 modulation to other NK cell receptors, we additionally stained cells for their cell surface expression using anti-CD226, anti-CD155, and anti-NTB-A–specific antibodies (*9*, *11*, *40*, *41*). CD226 was not modulated by HIV-1 (Fig. 2C), whereas CD155 (Fig. 2D) and NTB-A (Fig. 2E) were down-regulated upon HIV-1 infection, also dependent on the concerted action of Vpu and Nef. However, HIV-1–infected cells still retained about 75% of CD155 and NTB-A surface expression, whereas only ~40% of CD96 remained on the cell membrane of HIV-1–infected CD4^+^ T cells.

In addition, we assessed whether primary HIV-1 strains derived from people living with HIV-1 (PLWH) are also capable to modulate CD96. For this, we used two strains obtained during acute HIV-1 infection that harbored also inactivating mutations in *vpu* and *nef*, alone or in combination. CH058 and CH077 reduced CD96 cell surface levels to a similar extent as compared to HIV-1 NL4-3, our HIV-1 reference strain (Fig. 2F). In addition, the relevance of Nef for this phenotype was again inferior to that of Vpu, while inactivating both viral accessory proteins resulted in a complete loss of HIV-1’s activity to interfere with CD96 surface expression, also in those primary strains. Together, CD96 is down-regulated from the cell surface of HIV-1–infected CD4^+^ T cells by Nef and Vpu, and the extent of CD96 downmodulation by HIV-1 is more pronounced as compared to CD155 and NTB-A.

### HIV-1 Nef and Vpu target CD96 for lysosomal degradation

**Fig. 3.**
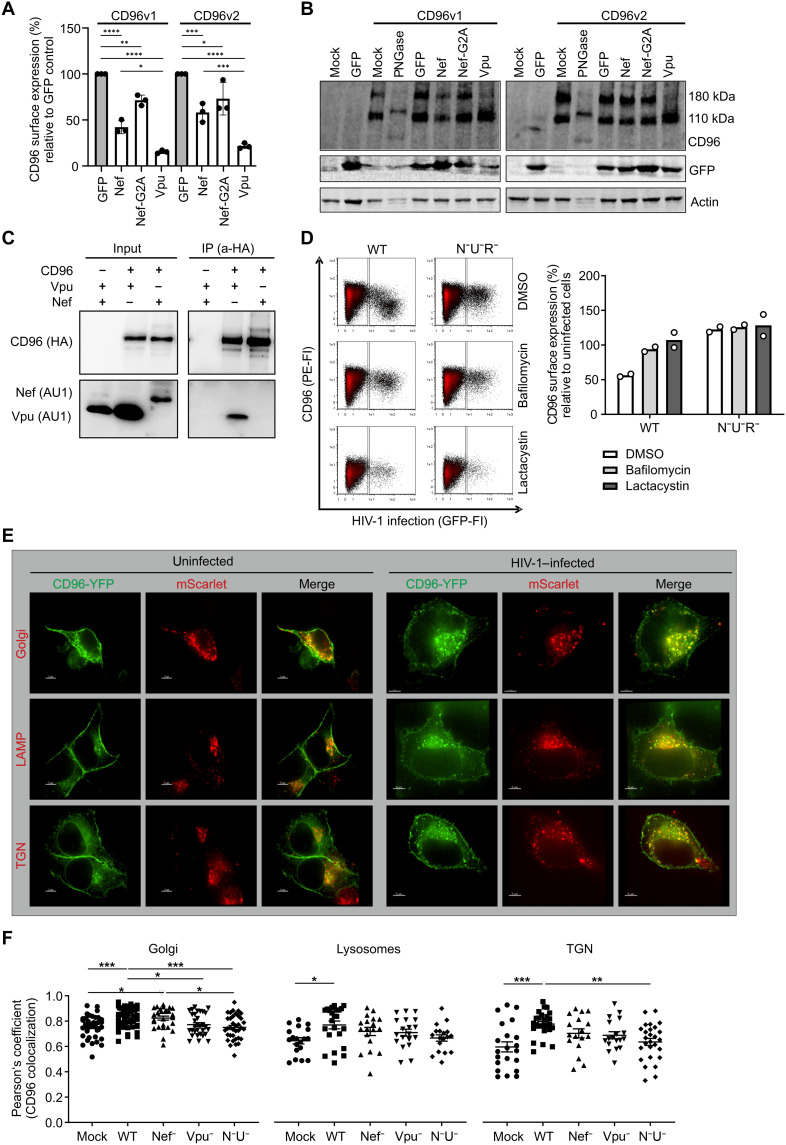
CD96 is down-regulated by HIV-1 Nef and Vpu involving lysosomal degradation and intracellular sequestration. 293T cells were transfected to express CD96v1, CD96v2, and either NL4-3 Vpu, NA7 Nef, GFP, or the Nef-G2A mutant. At 36 hours, cells were (**A**) harvested to measure cell surface CD96 via flow cytometry (*n* = 3) or (**B**) lysed for Western blot (WB) analysis with the indicated antibodies. (**C**) 293T cells were transfected to express HA-tagged CD96 and AU1-tagged Nef or Vpu. At 24 hours, cells were lysed in 0.5% Brij 98 immunoprecipitation buffer. CD96-HA and AU1-tagged Nef and Vpu were analyzed in input lysates and immunoprecipitates (IP) by anti-HA (a-HA)– and anti-AU1–specific antibodies. (**D**) Primary CD4^+^ T cells were infected with HIV-1 NL4-3–IRES–eGFP containing the indicated deletions in *vpr*, *nef*, and *vpu*. Five hours postinfection, cells were treated with 100 nM bafilomycin A1 (BafA1), 10 μM lactacystin (LC), or dimethyl sulfoxide (DMSO). Twenty-four hours postinfection, cells were harvested and analyzed for cell surface CD96 by flow cytometry. The CD96 MFI was used to calculate CD96 surface expression of HIV-1–infected cells compared to uninfected cells. (**E**) HeLa cells were transfected to express CD96v2–yellow fluorescent protein (YFP) and the indicated pmScarlet vectors to label cellular compartments. Twenty-four hours after transfection, cells were infected with vesicular stomatitis virus glycoprotein–pseudotyped HIV-1 NL4-3–IRES–mTagBFP reporter virus with deletions in *nef* and *vpu*. Twenty-four hours postinfection, cells were imaged via confocal microscopy. (**F**) CD96 colocalization within the indicated compartments was quantified using Pearson’s correlation coefficient. Representative confocal images of *n* = 17 to 48 analyzed cells were plotted (means ± SEM). Significance was tested using a one-way ANOVA with Tukey’s multiple comparison test. ****P < 0.0001, ***P < 0.001, **P < 0.01, and *P < 0.05.

To elucidate the mechanism behind the down-regulation of CD96, we next examined whether Vpu and Nef affect total CD96 steady-state levels. For this, 293T cells were transfected to express CD96v1 or CD96v2 along with Nef or Vpu, and then cell surface CD96 was analyzed by flow cytometry (Fig. 3A), and total CD96 steady-state levels were analyzed by immunoblotting (Fig. 3B). Our flow cytometry analysis showed that, also in the context of co-transfection in 293T cells, Nef and Vpu alone are sufficient to down-regulate cell surface CD96 (Fig. 3A). Nef reduced total levels of CD96v1, whereas it had no detectable effect on CD96v2. In contrast, Vpu reduced the higher–molecular weight band of both CD96v1 and CD96v2 (Fig. 3B). This effect is not related to interference with CD96 glycosylation by Nef or Vpu, since treatment with peptide *N*-glycosidase F (PNGase F) alters the migration pattern of both CD96 bands.

Next, we investigated whether Nef or Vpu directly interacts with CD96. For this, 293T cells were transfected to express hemagglutinin (HA)–tagged CD96 and AU1-tagged Nef or Vpu. Twenty-four hours posttransfection, cells were lysed, and CD96 was immunoprecipitated using an anti-HA–specific antibody, and input lysates and immunoprecipitates (IPs) were separated via SDS–polyacrylamide gel electrophoresis (PAGE) (Fig. 3C). CD96, Nef, and Vpu were detected using anti-HA– and anti-AU1–specific antibodies, respectively. Both Nef and Vpu were detected in the input lysates, but only Vpu coimmunoprecipitated with CD96, indicating that Vpu, but not Nef, might be able to directly bind CD96.

Nef and Vpu can serve as adaptors to target cellular proteins, potentially also CD96, for proteasomal or lysosomal degradation. To test this hypothesis, we treated primary CD4^+^ T cells infected with HIV-1 NL4-3–IRES–eGFP reporter virus, or the N^−^U^−^R^−^ mutant virus, with bafilomycin A1 (BafA1), a lysosomal inhibitor, or lactacystin (LC), a proteasome inhibitor. Five hours postinfection, cells were treated with either inhibitor, and 24 hours later, CD96 down-regulation was assessed in the HIV-1–infected (eGFP^+^) population of cells (Fig. 3D). Upon inhibition of the lysosomal pathway via BafA1, HIV-1 was not able to down-regulate CD96. Although treatment with LC also impaired the ability of HIV-1 to down-regulate CD96 from the cell surface, it is difficult to clearly conclude on this, since LC interfered with productive HIV-1 infection, was slightly cytotoxic, and reduced CD96 cell surface levels also in uninfected CD4^+^ T cells (Fig. 3D). In conclusion, HIV-1–mediated down-regulation of CD96 from the cell surface involves lysosomal degradation of the receptor.

To address whether Nef and Vpu might additionally interfere with subcellular CD96 trafficking and localization, we transfected HeLa cells to express yellow fluorescent protein (YFP)–tagged CD96v2 and markers of cellular organelles fused to mScarlet. By this, CD96 localization and association with the Golgi, lysosome-associated membrane protein (LAMP), and the trans-Golgi network (TGN) were visualized. Twenty-four hours posttransfection, cells were infected with HIV-1 NL4-3–IRES–mTagBFP reporter virus also harboring inactivating mutations in Nef, Vpu, or both viral proteins (N^−^U^−^). Twenty-four hours postinfection, cells were imaged via confocal microscopy (Fig. 3E), and CD96 colocalization within the indicated compartments was quantified (Fig. 3F). This analysis revealed that, in uninfected cells, CD96 mainly localizes to the plasma membrane but is also detected in the Golgi, lysosomes, and the TGN. However, upon infection with HIV-1, as expected, CD96 was less prominent at the cell surface but rather sequestered to internal compartments including the Golgi, TGN, and lysosomes (Fig. 3F). This intracellular sequestration of CD96 was dependent on functional expression of Nef and Vpu, since deletion of both proteins resulted in the same cellular distribution of CD96 as compared to uninfected cells.

Together, our mechanistic studies revealed that Nef and Vpu affect total CD96 steady-state protein levels and its cell surface expression. Both viral proteins interfere with intracellular trafficking of CD96 and sequester the receptor into the lysosomal compartment for targeted degradation.

### CD96 downmodulation is conserved among HIV-1 Nef and Vpu alleles and functionally distinct from modulation of CD4 and CD317

**Fig. 4.**
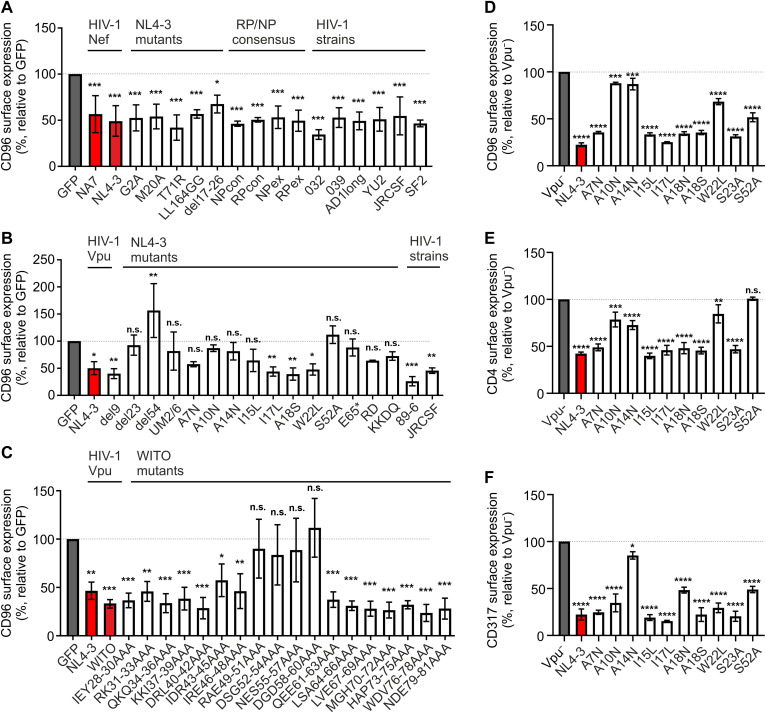
Analysis of CD96 cell surface modulation by Nef and Vpu variants. (**A** to **C**) Jurkat-TAG cells stably expressing CD96v2 were electroporated to express the indicated Nef (A), Vpu (B), and WITO Vpu (C) variants. Twenty-four hours postelectroporation, cells were harvested and stained for cell surface CD96 and analyzed by flow cytometry. The CD96 receptor expression was calculated relative to the GFP-only–expressing negative control, which is marked in gray. Reference NA7 and NL4-3 Nef and Vpu proteins are marked in red. (**D** to **F**) Primary CD4^+^ T cells were infected with *nef*-defective HIV-1 NL4-3 Gag-iGFP expressing the indicated Vpu variants. Forty-eight hours postinfection, cells were harvested and stained for cell surface CD96 (D), CD4 (E), and CD317 (F) and analyzed by flow cytometry. The receptor expression of infected cells was calculated relative to the negative control GFP (Vpu^−^). Values from three independent experiments were plotted (means ± SD). Significance was tested using a one-way ANOVA with Dunnett’s multiple comparison test. ****P < 0.0001, ***P < 0.001, **P < 0.01, and *P < 0.05. n.s., not significant.

To identify possible structure-function relationships, 5 Nef and 35 Vpu mutants harboring site specific mutations or deletions and 10 primary Nef and 3 Vpu alleles were screened for their capability to downmodulate CD96. For this, we generated Jurkat T cells stably expressing CD96v2 and electroporated them to express the indicated Nef (Fig. 4A), Vpu (Fig. 4B), or WITO Vpu (Fig. 4C) variants. Twenty-four hours postelectroporation, cells were analyzed for CD96 cell surface expression by flow cytometry. CD96 receptor expression was calculated relative to a GFP-only–expressing control (Fig. 4).

All HIV-1 Nef variants (NA7, NL4-3, YU2, SF2, and JR-CSF), NL4-3 Nef mutants (G2A, M20A, T71R, LL164GG, and del17-26), the consensus HIV-1 Nef alleles obtained from nonprogressors (NPcon and NPex) and rapid progressors (RPcon and RPex), and the primary HIV-1 Nef alleles AD-93 (AD1long), 032an-93 (032Nef), and 039 nm-94 (039Nef) were able to markedly reduce CD96 cell surface levels (Fig. 4A). Furthermore, HIV-1 Vpu NL4-3, JR-CSF, and 89-6 down-regulated CD96 (Fig. 4B). Deletion of the cytoplasmic domains (del23 and del54) completely abolished this function, whereas a deletion of only nine amino acids (del9) retained the ability to modulate CD96. Among the tested Vpu mutants I17L, A18S and W22L were able to down-regulate CD96 from the cell surface. In contrast, the other Vpu mutations seem to impair important structural motifs for this functionality, since they were attenuated or completely lost their ability to down-regulate CD96 (Fig. 4B). Furthermore, a set of HIV-1 WITO Vpu mutants confirmed the importance of domains between R49-D60 for CD96 downmodulation, including two serine residues at positions 53 and 57 known to represent a β-TrCP binding motif (Fig. 4C) (*42*).

Next, the ability of Vpu to down-regulate CD96 was analyzed in the context of HIV-1 infection and compared to other established Vpu functions such as the down-regulation of CD4 and CD317 (Tetherin). To do this, we generated the selected *vpu* mutants in an HIV-1 eGFP-expressing reporter virus lacking Nef-expression, hence allowing to specifically monitor effects exerted by Vpu. Primary CD4^+^ T cells were infected with the NL4-3 HIV-1 reporter virus harboring the indicated mutations in *vpu* (Fig. 4, D to F). Forty-eight hours postinfection, CD4^+^ T cells were harvested, and modulation of CD96 (Fig. 4D), CD4 (Fig. 4E) and CD317 (Fig. 4F) was analyzed by flow cytometry. In consistence with our findings in Jurkat T cells, not only A10N and A14N but also the W22L of the Vpu transmembrane domain seem involved in CD96 modulation, with moderate impairment by S52A (Fig. 4D). In contrast, Vpu-mediated CD4 downmodulation (Fig. 4E) was most severely impaired by S52A. Moreover, A10N and W22L in Vpu were dispensable for CD317 down-regulation, with most disrupting functional impairment imposed by A14N (Fig. 4F). These findings suggest that, although structural requirements for Vpu to downmodulate CD96, CD4, and CD317 overlap, specific amino acids exhibit differential importance for each function, highlighting that these processes can be functionally separated.

### CD96 does not alter HIV-1 replication in primary CD4^+^ T cells nor cell-mediated cytotoxicity

**Fig. 5.**
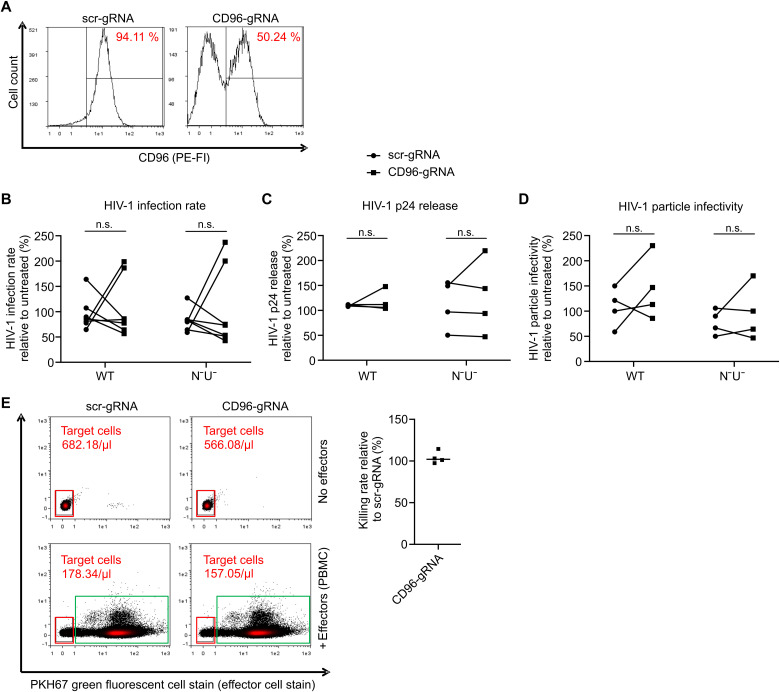
CD96 in primary CD4^+^ T cells does not modulate HIV-1 replication or cellular cytotoxicity. CD96 was knocked out in stimulated primary CD4^+^ T cells by CRISPR-Cas9. (**A**) Three days later, the KO was confirmed by CD96 cell surface staining, and (**B** to **D**) cells were infected with equal p24 amounts of HIV-1 NL4-3–IRES–eGFP with or without deletions in *nef* and *vpu*. (B) Forty-eight hours postinfection, cells were analyzed for the rate of productive infection by their GFP expression. (C) In addition, supernatant was collected to quantify for HIV-1 production and release via HIV-1 p24 ELISA and (D) determine the infectivity of released HIV-1 particles via reinfection of Jurkat cells with supernatants normalized for HIV-1 p24. Values from four independent experiments were plotted (means ± SD). Significance was tested using a two-way ANOVA with Sidak’s multiple comparison test. (**E**) CD96 KO CD4^+^ T cells were used as target cells and cocultured with PKH67-stained PBMCs, which served as effector cells for the cellular cytotoxicity assay. The coculture was incubated for 16 hours at an effector:target cell ratio of 40:1. Cell count of target cells was analyzed by flow cytometry. Killing efficiency was calculated by dividing the cell counts of target cells in the absence of effector cells though the cell counts in the presence of effectors relative to mock. Values from four independent experiments were plotted.

To functionally evaluate the role of CD96 in HIV-1–infected CD4^+^ T cells, we established CRISPR-Cas9–mediated gene knockout (KO) electroporating assembled Cas9 ribonucleoprotein (RNP) complexes in phytohemagglutinin (PHA)/IL-2–stimulated primary CD4^+^ T cells (*43*, *44*). Using this system, we achieved a KO efficiency of 50 to 80% across various donors (see example Fig. 5A). Control and CD96 RNP-electroporated cells were infected with HIV-1 NL4-3–IRES–eGFP and a mutant that does not express functional Vpu or Nef. Forty-eight hours postinfection, cells were analyzed via flow cytometry for eGFP expression to determine the rate of productively HIV-1–infected primary CD4^+^ T cells (Fig. 5B). Overall, CD96 did neither influence WT HIV-1 infection rate nor that of the *vpu*- and *nef*-deleted virus devoid of active CD96 down-regulation. In addition, cell culture supernatants were quantified for HIV-1 p24 production and release by enzyme-linked immunosorbent assay (ELISA) (Fig. 5C), revealing that CD96 does not affect viral particle production or release. Moreover, aliquots of the same supernatants were used to infect Jurkat cells, allowing us to quantify the infectivity of released viral particles (Fig. 5D). This indicates that expression levels of CD96 in virus-producing cells do not alter the infectivity of HIV-1 particles. Together, CD96 expression on infected primary CD4^+^ T cells has no detectable effect on virus replication in this system.

CD96 is involved in NK cell–mediated cytotoxicity (*28*), which is why we hypothesized that HIV-1 might down-regulate CD96 to interfere with this process. To test this, we cocultured primary PHA-activated CD4^+^ T cells (with or without CD96 KO) with autologous peripheral blood mononuclear cells (PBMCs) as effector cells. Given that CD96 has been suggested to act as inhibitory NK cell receptor (*28*), we hypothesized that CD96 KO cells will be more efficiently killed by the NK cell population within the PBMCs. Before coculture, the effector PBMCs were stained with a cell tracker, allowing us to discriminate them from the CD4^+^ target cells (Fig. 5E; target cells are marked by the red square, and the cocultured and labeled effector cells, which exert green fluorescence, are marked by the green square). After 24 hours of coculture, we quantified the absolute number of remaining target cells per microliter of sample fluid and calculated the killing rate by dividing the target cell number without effector cells by the target cell number in the presence of effector cells [Fig. 5E; exemplary numbers are indicated in the primary fluorescence-activated cell sorting (FACS) plots]. This analysis revealed that KO of CD96 did not affect the killing rate efficiency (Fig. 5E, right), indicating that in this experimental system, CD96 cell surface expression does not influence NK cell–mediated cytotoxicity in primary CD4^+^ T cells.

### CD96^Hi^ CD4^+^ T cells exhibit a T helper 1/T helper 17–like phenotype

**Fig. 6.**
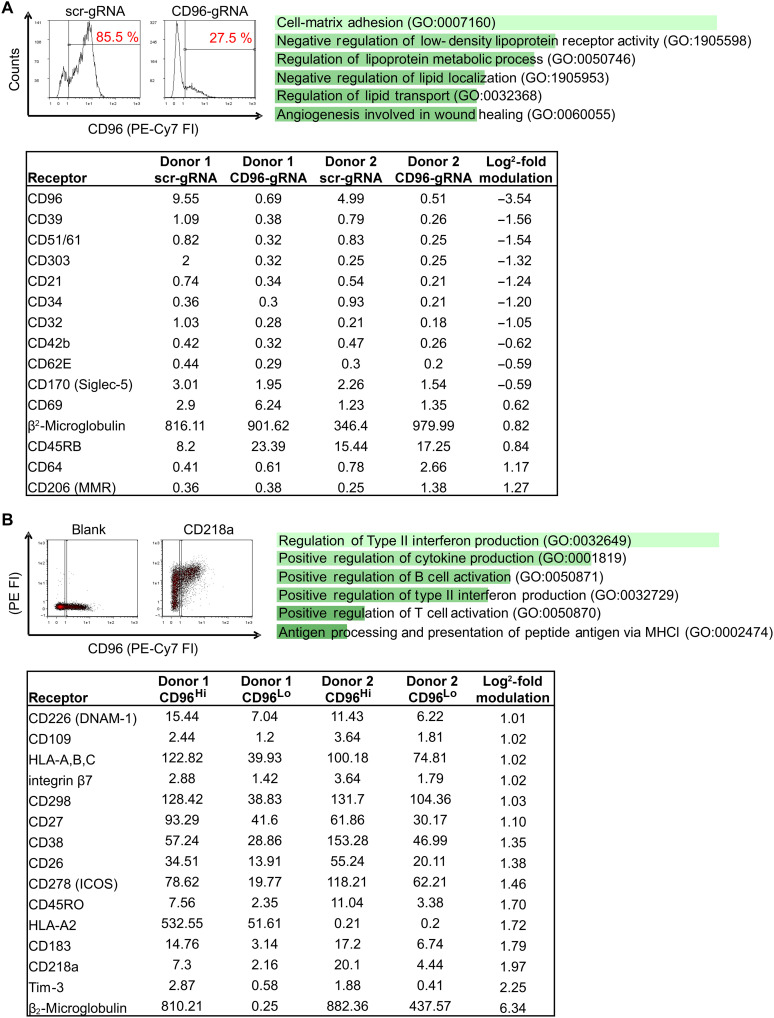
CD96-associated cell surface receptor expression profile of primary CD4^+^ T cells. Primary CD4^+^ T cells from two donors were stimulated for 3 days with IL-2 and PHA (**A**) before CRISPR-Cas9–mediated KO of CD96. After confirmation of the CD96 KO by flow cytometry, CD96 KO cells and scr-RNA control cells were stained with 332 PE-conjugated antibodies of the LegendScreen kit and analyzed by flow cytometry. MFIs of all receptors were compared between CD96 KO and scr-RNA control cells, and the log_2_-fold modulation of receptors was quantified. Receptors with a log_2_-fold modulation of <−0.58 or >0.58 on CD96 KO cells compared to scr-RNA cells are summarized in the table. These receptors were analyzed via Enrichr and are linked to the shown biological pathways (adjusted *P* value for all pathways < 0.05). The underlying raw data are included in dataset S2. GO, gene ontology. (**B**) Stimulated primary CD4^+^ T cells were stained with anti-CD96–PE/Cy7–specific antibody and 332 PE-conjugated antibodies of the LegendScreen kit and analyzed via flow cytometry. MFIs of all receptors were compared between CD96^Hi^- and CD96^Lo^-gated CD4^+^ T cells (see exemplary FACS plot), and the log_2_-fold modulation of receptors was quantified. Receptors which are >1.00 log_2_-fold higher expressed on CD96^Hi^ cells compared to CD96^Lo^ cells are summarized in the table. These receptors were analyzed via Enrichr and are linked to the shown biological pathways (adjusted *P* value for all pathways < 0.05). The underlying raw data are included in dataset S4.

To gain additional insights into the role of CD96 in primary CD4^+^ T cells, we profiled CD96 KO cells for cell surface receptor expression using the same flow cytometry–based screen used to identify HIV-1–modulated receptors in this study and before to phenotype receptor expression upon CD4^+^ T cell stimulation (*45*). For the 332 PE-conjugated antibodies included in the screen, the PE median fluorescence of CD96 KO CD4^+^ T cells was compared to control cells and used to calculate the X-fold difference in receptor expression upon CD96 KO (Fig. 6A). Although CD96 expression was nearly fully diminished, there was very high variation in total surface expression of the various receptors between the two tested donors. We listed receptors with a log_2_-fold receptor modulation of <−0.58 or >0.58, meaning that they are expressed more than 1.5-fold higher or lower, respectively, on CD96 KO cells (Fig. 6A and dataset S2 for the list of all receptors). These identified receptors were analyzed for common pathways via Enrichr (https://maayanlab.cloud/Enrichr). Biological processes related to the differentially expressed genes in the CD96 KO cells pointed toward a role for CD96 in cellular adhesion. On a molecular level, this was reflected by reduced expression of integrins (CD51/61) and the adhesion molecule CD170 (Siglec-5) (Fig. 6A).

Given the high variation in cell surface receptor expression upon RNP electroporation in primary CD4^+^ T cells, we transitioned to more consistent systems that would reduce technical perturbations. For this, we used the CD4^+^ T cell line SupT1 that expresses low levels of CD96 and generated CD96 KO cells via lentiviral transduction using the CRISPR-Cas9 system. The CD96 KO was verified by flow cytometry (fig. S2A), and, subsequently, cells were subjected to RNA sequencing (RNA-seq) analysis to reveal host cell pathways that are interrogated upon CD96 depletion (dataset S3). As expected from the low expression of CD96 in this immortalized T cells, the transcriptomic changes we witnessed were subtle (fig. S2B). Quantitative assessment revealed modulation of 22 genes according to an adjusted *P* < 0.1 (fig. S2C) that showed significant association with immune regulatory pathways, for instance, the PD-1 checkpoint activation, T helper 1 (T_H_1)/T_H_2, and T_H_17 T cell differentiation (fig. S2D). To corroborate this functional association with phenotypic markers again in a more relevant system, we used activated CD4^+^ T cells, the primary targets of HIV-1 infection. Primary CD4^+^ T cells were costained with anti-CD96–PE/Cy7–specific antibody and the 332 PE-conjugated antibodies and analyzed by flow cytometry. This approach allowed us to categorize the primary CD4^+^ T cell population into CD96^Hi^ versus CD96^Lo^ cells, associating distinct cell surface expression patterns with CD96 expression levels (Fig. 6B, showing receptors with a median fluorescence intensity of >1.00 and a mean log_2_-fold difference in receptor expression of >1.00, and dataset S4 for the list of all receptors). Enrichment analysis revealed that biological processes associated with high CD96 levels expression included type II interferon production, positive regulation of immune cell activation, and cytokine production. At the molecular level, CD96^Hi^ cells were markedly enriched for HLA-associated receptors (HLA-A, HLA-B, and HLA-C; HLA-A2 and β_2_-microglobulin) in addition to Tim-3, CD218a (IL-17RA), CD183 (CXCR3), and the costimulatory CD278 (ICOS) (Fig. 6B). Notably, the latter receptors are highly expressed on T_H_1/T_H_17-polarized CD4^+^ T cells, which play a crucial role in regulating antiviral immune responses (*46*–*48*). Furthermore, the phenotypic screen in primary CD4^+^ T cells converges with and confirms the RNA-seq data obtained from CD96 KO SupT1 CD4^+^ T cells (fig. S2). Thus, CD96^Hi^ expression on CD4^+^ T cells may represent a marker for activated T_H_1/T_H_17 cells, and CD96 seems functionally involved in regulating T_H_1/T_H_17 T cell differentiation.

### CD96^Hi^ CD4^+^ T cells secrete high levels of interferon-γ/IL-17 and exert altered migratory behavior in the medaka in vivo model

**Fig. 7.**
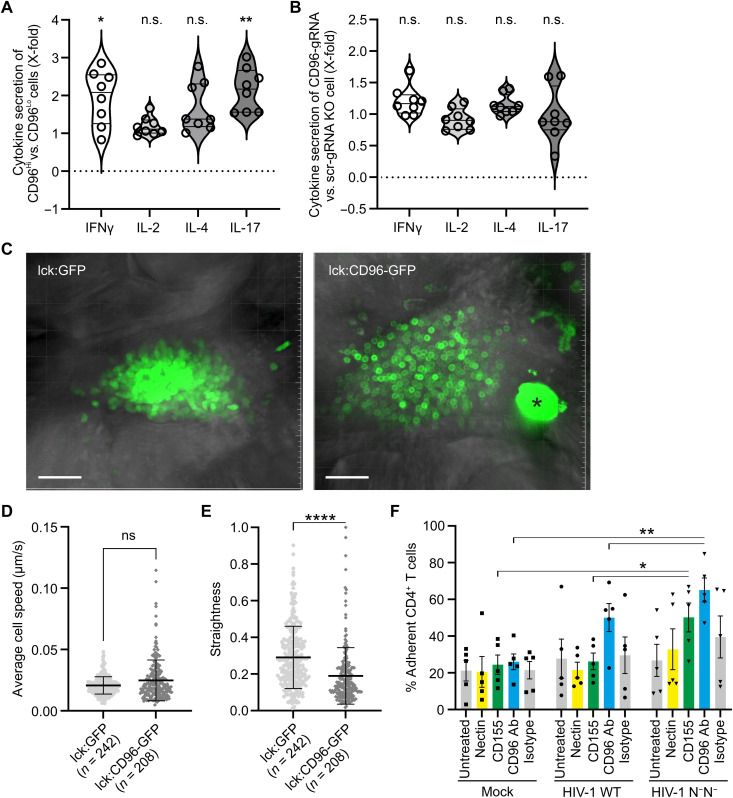
Functionality of CD96 in CD4^+^ T cells and HIV-1 infection. Primary CD4^+^ T cells were IL-2/PHA stimulated for 3 days and left untreated (**A**) or subjected to CRISPR-Cas9–mediated CD96 KO (**B**). Twenty-four hours later, cells were restimulated with anti-CD3/anti-CD28, and, 48 hours later, cells were analyzed for cytokine secretion and costained for CD96. Cytokine secretion of (A) CD96^Hi^ cells was compared to CD96^Lo^ cells and (B) that of CD96 KO cells to scr-RNA control cells (*n* = 8, means ± SEM, two-way ANOVA with Sidak’s multiple comparison). **P < 0.01 and *P < 0.05. (**C**) An hCD96-GFP fusion protein is predominantly localized on the cell membrane of medaka thymic T cells (movie S1) compared with GFP-only–expressing controls (movie S2). Scale bars, 30 μm. Average cell speed (**D**) and straightness (**E**) of GFP^+^ cells in the thymus. *N* = total number of cells examined from >3 samples per condition (means ± SEM, two-tailed Mann-Whitney test). ****P < 0.0001. The asterisk (*) marks an autofluorescent pigment cell cluster. (**F**) Primary CD4^+^ T cells prestimulated with CD3/CD28 for 3 days were infected with HIV-1 NL4-3–IRES–eGFP or the N^−^/U^−^ variant. Forty-eight hours postinfection, cells were seeded onto 96-well plates coated with CD96 ligands Nectin-1, CD155, and anti-CD96 or an isotype control. After 45 min at 37°C, nonadherent and adherent cells were collected separately and quantified by flow cytometry. Adhesion was calculated as the percentage of adherent cells relative to the total number of cells (*n* = 5, means ± SEM, paired two-way ANOVA with Fisher’s least significant difference). **P < 0.01 and *P < 0.05.

We next followed up on the hypothesis that CD96^Hi^ CD4^+^ T cells may represent a cell population functionally polarized toward a T_H_1/T_H_17-like phenotype. For this, we profiled cytokine secretion using a flow cytometry–based assay, allowing us to correlate cytokine release with CD96 expression. Notably, activated CD96^Hi^ CD4^+^ T cells showed a significant increase in IFN-γ and IL-17 release compared to CD96^Lo^ CD4^+^ T cells (Fig. 7A). In contrast, cytokine secretion in CD96 KO CD4^+^ T cells showed no differences (Fig. 7B).

To explore the importance of CD96 in vivo, we used the medaka fish model, which is a model organism for studying the behavior of T cells during their development. Medaka share key molecular and cellular features of T cell development with mammals (*49*–*52*). In addition, their small size, ease of handling and optical transparency, make them an ideal model for studying cellular dynamics within the entire thymus organ noninvasively, enabling the analysis of the consequences of hCD96 expression in a complex in vivo system (*49*, *52*). To achieve T cell–specific expression of CD96 in medaka, we used a CD96v2-GFP construct that drives hCD96 expression under control of the medaka thymocyte-specific lck promoter (lck:CD96-GFP). The construct was then injected into the one-cell stage embryos, and GFP was monitored in the thymus during the embryonic development using confocal microscopy. The medaka lck:GFP transgenic line (*50*) was used as a control. As expected, CD96 localized to the cell membrane in T cells within the thymus of transgenic fish, whereas GFP was distributed throughout the cells in the control group (Fig. 7C). Next, we performed three-dimensional (3D) time-lapse in vivo recording of the thymus and used the image data to assess the velocity and straightness of T cells in the embryonic thymus, as described previously (*50*). While the average cell speed did not differ significantly between CD96-GFP–overexpressing T cells and GFP-only–expressing cells (Fig. 7D), the straightness of CD96-expressing T cells was significantly reduced (Fig. 7E), indicating that CD96 may functionally interfere with cellular migration. To corroborate this finding in the context of human T cell biology and HIV-1 infection, we established a T cell adhesion assay. CD4^+^ T cells infected with either WT HIV-1 or a mutant virus lacking functional Vpu and Nef expression were allowed to adhere to potential CD96 ligands, Nectin-1 (*53*) and CD155 (*54*), as well as to a CD96-specific antibody used as a positive control (Fig. 7F). Overall, HIV-1–infected CD4^+^ T cells tend to be more adherent compared to mock-infected cells, and, notably, even WT HIV-1 showed increased adherence to CD96 antibody. We did not observe enhanced adherence to coated Nectin-1. In contrast, while WT HIV-1 or mock-infected CD4^+^ T cells showed similar adherence to CD155 (~20% of cells), 50 to 60% of CD4^+^ T cells infected with *vpu*/*nef*-defective HIV-1 exhibited CD155 adherence. Similarly, more HIV-1–infected CD4^+^ T cells adhered to CD96 antibody in the absence of functional Vpu and Nef expression (Fig. 7F). Together, this indicates that downmodulation of CD96 on HIV-1–infected CD4^+^ T cells by Vpu and Nef functionally impairs cellular interaction with the natural CD96 ligand CD155 and with a CD96-specific antibody.

In conclusion, CD4^+^ T cells expressing high levels of CD96 show phenotypic markers and exhibit a functional cytokine secretion profile reminiscent of T_H_1/T_H_17-like CD4^+^ T cells. In addition, T cells expressing hCD96 displayed altered migratory behavior in our in vivo model, and HIV-1–infected CD4^+^ T cells showed reduced interaction and adherence to CD96 ligands.

### CD96 stimulation induces secretion of T_H_1/T_H_17 effector cytokines

**Fig. 8.**
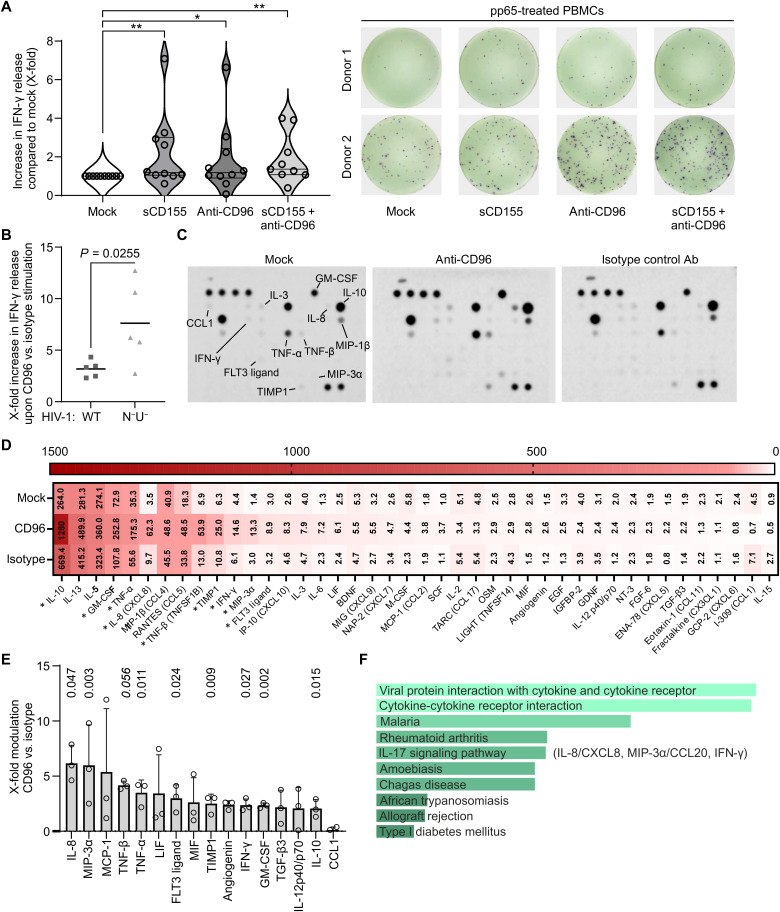
CD96 ligation induces release of antiviral and proinflammatory cytokines. (**A**) PBMCs were stimulated with HCMV pp65 peptide and costimulated either alone or in combination with CD155 or anti-CD96 (each 5 μg/ml) and stained for IFN-γ 21 hours later. IFN-γ secretion was calculated as fold change relative to pp65 stimulation only (mock). (*n* = 10, Kruskal-Wallis test with uncorrected Dunn’s test). *P < 0.05 and **P < 0.01. Depicted are primary data from two donors. (**B**) Resting primary CD4^+^ T cells were infected with HIV-1 NL4-3–IRES–eGFP or the N^−^/U^−^ variant and stimulated at 6 hours postinfection with CD3/CD28 antibodies. Two days postinfection, cells were restimulated with CD3/CD28 antibodies either alone or in combination with anti-CD96 or an isotype control. Three days later, IFN-γ secretion was assessed via flow cytometry (*n* = 5, paired one-tailed *t* test). (**C**) Primary CD4^+^ T cells were stimulated with CD3/CD28 antibodies (0.5 μg/ml each) and mock treated or costimulated with coated CD96 antibody (2.5 μg/ml) or isotype (2.5 μg/ml). Three days poststimulation, supernatants were harvested for a cytokine array (*n* = 3, one representative array shown). GM-CSF, granulocyte-macrophage colony-stimulating factor. (**D**) Heatmap illustrating relative abundance of analyzed cytokines normalized to the respective controls. [Mean values, *n* = 3; *n* = 2 for IL-6, macrophage colony-stimulating factor (M-CSF), and fibroblast growth factor 6 (FGF-6)]. Only cytokines with >2% relative abundance in at least one treatment condition were included. LIF, leukemia inhibitory factor; BDNF, brain-derived neurotrophic factor; SCF, stem cell factor; MIF, migration inhibition factor; EGF, epidermal growth factor; GDNF, glial cell line–derived neurotrophic factor. (**E**) X-fold change in cytokine secretion between CD96 antibody versus isotype-treated CD4^+^ T cells was calculated, and the waterfall plot depicts changes of >2 (*n* = 3, ±SD, multiple unpaired *t* test with individual *P* values). (**F**) Pathway analyses using Enrichr (Kyoto Encyclopedia of Genes and Genomes 2021 database) and as input the nine significantly regulated genes from (E). Bar length and brightness correlates with significance (*q* > 0.0001). For “IL-17 signaling,” key cytokines from (E) are indicated.

Thus far, we characterized CD96 functionality using CRISPR-Cas9–based genetic CD96 KO in conjunction with phenotypic profiling of CD96^Hi^ versus CD96^Lo^ CD4^+^ T cells. However, this analysis precludes a scenario in which the receptor is triggered in the immunological context via its ligand CD155 or an agonistic antibody. To address this, we first examined whether CD96 ligation might enhance release of the antiviral type II interferon and T_H_1 cytokine IFN-γ, as suggested by our RNA-seq profiling (fig. S2) and phenotypic T cell characterization (Figs. 6B and 7A). We incubated PBMCs from human cytomegalovirus (HCMV)–positive blood donors with the HCMV peptide pp65 to induce a T cell response and measured IFN-γ release using an enzyme-linked immunospot (ELISpot) assay (Fig. 8A). Notably, upon costimulation of PBMCs with soluble CD155, the ligand of CD96, or an antibody known to bind CD96, we observed up to an eightfold increase in IFN-γ release from peptide-loaded PBMCs (Fig. 8A). While these effects showed statistical significance, pronounced donor-dependent variations were detectable, presumably due to individual differences in CD96 levels, an observation we already made in the context of our initial screen for HIV-1–modulated cell surface receptors (dataset S1). Similarly, in the context of HIV-1–infected primary CD4^+^ T cells, CD96 stimulation triggered the release of IFN-γ. Notably, this induction of IFN-γ was, on average, 4.4-fold higher when cells were infected with* nef*- and *vpu*-defective HIV-1 (N^−^U^−^), which lacks the ability to downmodulate cell surface CD96 (Fig. 8B). On a broader scale, in response to CD96 stimulation with an antibody, activated primary CD4^+^ T cells showed increased secretion of several cytokines (Fig. 8, C and D). Our data revealed a significant enrichment in the release of proinflammatory cytokines including IL-8, macrophage inflammatory protein–3α (MIP-3α) (CCL20), tumor necrosis factor–β (TNF-β), TNF-α, and IFN-γ (Fig. 8E). Notably, the latter are involved in the antiviral immune response and represent effector cytokines of IL-17 signaling (*55*) and T_H_1/T_H_17 T cell functionality (Fig. 8, E and F). Together, the data show that ligation of CD96 on primary CD4^+^ T cells via its natural ligand sCD155 or CD96-specific antibodies triggers the release of highly potent antiviral effector cytokines and mounts a type II interferon response, which is antagonized by HIV-1 Nef and Vpu.

## DISCUSSION

In this study, by broad assessment of HIV-1–induced plasma membrane alterations, we identified a previously unidentified mechanism of how HIV-1 manipulates virally infected primary CD4^+^ T cells. We found that HIV-1 robustly down-regulates CD96 from the surface of infected CD4^+^ T cells. We define the mechanism of CD96 modulation as the concerted action of the viral accessory proteins Nef and Vpu that interfere with CD96 trafficking and target the receptor for degradation. Our data demonstrate that this is a conserved and independent function of several primary HIV-1 Nef and Vpu alleles and CD96 downmodulation is also observable upon infection with primary patient-derived HIV-1 variants. Through the results presented herein, we shed light on the poorly understood role of CD96 in human immunobiology in the context of primary CD4^+^ T cells. Our data support a direct role of CD96 in mounting an antiviral T_H_1/T_H_17 CD4^+^ T cell immune response and the production of proinflammatory and antiviral cytokines including TNF-β, TNF-α, and the type II interferon. Furthermore, in vivo, CD96 modulates migratory behavior of CD4^+^ T cells. Together, by our work, we reveal the functionality and importance of CD96 on primary CD4^+^ T cells and found a previously unidentified strategy how HIV-1 evades the antiviral immune response.

Given the fact that manipulation of cell surface receptors by HIV-1 is a long-known strategy of the virus to evade the innate and adaptive antiviral immune response (*2*, *4*, *56*), it might seem unexpected that down-regulation of CD96 in HIV-1–infected cells has not been revealed up to now. Notably, most in vitro infection studies and associated screening approaches are initiated using cell lines (*15*, *16*), as they are easier to handle and not affected by donor variability, as compared to primary cell systems in general and human CD4^+^ T cells in particular. In this context, it is noteworthy that, at least in the T cell lines tested in the course of our study, we did not detect robust CD96 expression on immortalized CD4^+^ T cell lines [e.g., low levels on Jurkat-TAG (Fig. 4) or SupT1 (fig. S2)]. This might explain why previous screens failed to identify CD96 as receptor manipulated by HIV-1 or its accessory proteins Nef and Vpu. Considering the latter, we postulate that the comprehensive “membranous fingerprint” of human primary CD4^+^ T cells, infected with replication competent HIV-1 provided here, represents a valid basis to focus on highly relevant surface receptors affected by productive HIV-1 infection. Of course, this conclusion has to be accompanied by stating the limitations of our approach: (i) The screen is not comprehensive as we can only detect cell surface receptors that are covered by the antibody panel provided in the screen; (ii) it is further limited to receptors expressed in the context of stimulated CD4^+^ T cells, missing receptors exclusively present on other HIV-1–relevant target cells in vivo, as for instance macrophages; (iii) there is no dynamic view on receptor modulations as we have only analyzed 48 hours postinfection; and (iv) the screen is conducted with a laboratory-adapted strain HIV-1 NL4-3, hence failing to detect receptor modulations that might occur only in the context of primary HIV-1 strains. On the other hand, we independently identify several surface receptors that were previously found to be down-regulated by HIV-1 in cell lines and primary CD4^+^ T cells. These are CD4; MHCI; CD317 (Tetherin) (*5*, *6*, *8*, *57*, *58*); the chemokine receptors CD197 (CCR7) (*35*), CD183 (CXCR3), and CD181 (CXCR1); (*36*), the IL-7RA (CD127) (*37*, *59*); and L-selectin (CD62L) (*39*). These findings prove the robustness of our screen and raise confidence in receptors identified by this approach.

Our analysis of CD96 downmodulation reveals that the protein is efficiently removed from the surface of infected CD4^+^ T cells. Similar to the downmodulation of CD4 and CD62L (*5*, *39*), this process involves more than one viral protein. HIV-1 Vpu and Nef interfere with intracellular trafficking and target the receptor for degradation. In addition, primary patient-derived Vpu and Nef alleles are able to down-regulate CD96. This is achieved in the context of a T cell line transfected to express the viral proteins and engineered for high levels of CD96 on the cell surface, demonstrating that Vpu and Nef alone are able to specifically target CD96. Our mutagenesis approach further revealed that overlapping, albeit not identical, motifs in Vpu are responsible for CD96 down-regulation and the modulation of CD4 and CD317, other bona fide targets of Vpu. This is in line with the concept of Vpu and Nef being viral adaptor proteins that use flexible surfaces to interact with host cell factors to target them for lysosomal or proteasomal degradation (*60*–*63*). Accordingly, mutation of the conserved Vpu β-TrCP–binding motif S52/S56 (*42*) disrupted down-regulation of CD96, CD4, and CD317. In contrast, mutation A10N in the Vpu-transmembrane domain allowed us to separate Vpu-mediated CD96 from CD317 down-regulation. Regions in Nef known to disrupt its ability to down-regulate MHCI (M20) (*64*) or CD4 (LL164) (*65*) did not impair down-regulation of CD96. Unexpectedly, Nef mutation G2A that interferes with Nef myristoylation and its membrane association (*66*) did not fully disrupt its ability to down-regulate CD96. In contrast, a deletion (del17-26) in the C-terminal region of Nef, known to be important for β-Cop binding (*67*), showed attenuated CD96-modulating activity. Hence, CD96 is robustly down-regulated by Vpu and Nef. This activity is independent of other Vpu and Nef functions, conserved upon primary HIV-1 alleles, and the sole expression of each of the viral proteins is sufficient to downmodulate the immune receptor. This suggests that there is a high evolutionary pressure for the virus to maintain this activity and to interfere with functional CD96 expression on the surface of virally infected CD4^+^ T cells.

So why is HIV-1 down-regulating CD96? Initially, CD96 was described as being part of a regulatory network of receptors on NK cells that engage its ligand CD155 (PVR) on target cells and activate or inhibit NK cells (*19*). In murine systems, CD96 is an inhibitory NK cell receptor. Therefore, it is proposed that CD96 might be an immunotherapeutic target unleashing the brakes from NK cells in addition to “classical” immunotherapy that acts on T cells (*30*–*32*, *68*). This is based on a landmark study that reported an inhibitory function for mCD96 in NK cells and mCD96 limiting the production of IFN-γ (*28*) and backed up by other studies demonstrating inhibitory roles of mCD96 on NK cells (*33*, *69*, *70*). However, in humans, CD96 is expressed not only on NK cells but also on T cells, is present in various splice forms, and contains additional intracellular motifs that resemble immunoreceptor tyrosine-based activation motifs, i.e., putative tyrosine-based activating motifs (*23*). Still, experimental evidence related to the importance of CD96 in humans is sparse, and recent work suggested a costimulatory function for hCD96 on CD8^+^ and CD4^+^ T cells (*29*, *71*), while other data also support an inhibitory function of hCD96 on human NK and T cells (*30*, *72*). We initially followed up on the hypothesis that CD4^+^ T cell–expressed CD96 might modulate NK cell activity, although it seemed counterintuitive that HIV-1 down-regulates a receptor that presumably acts inhibitory on attacking NK cells. Upon depletion of CD96 from ~50% of CD4^+^ T cells, we did observe neither increased nor decreased cell killing in our setup (Fig. 5E). In addition, depletion of CD96 from CD4^+^ T cells did not alter basic parameters of virus replication, i.e., infection rate, virus production, and HIV-1 infectious particle release, even when Nef and Vpu were inactivated rendering the virus inactive in CD96 down-regulation (Fig. 5, B to D). Therefore, we conclude that CD96 is dispensable for virus replication in cell culture settings in the absence of external immune control. Conversely, when we immunophenotyped CD4^+^ CD96^Hi^ T cells, we find strong enrichment in expression of Tim-3, CD218Aa (IL-17RA), CD183 (CXCR3), and costimulatory CD278 (ICOS) in addition to HLA molecules, all of which are markers for CD4^+^ T_H_1/T_H_17 cells that play a prominent role in the antiviral immune response (*46*–*48*). This phenotypic data are supported by four functional evidences: (i) elevated IFN-γ and IL-17 secretion from CD4^+^ CD96^Hi^ T cells (Fig. 6B), (ii) RNA-seq of CD96 KO SupT1 CD4^+^ T cells (fig. S2), (iii) CD96 stimulation via sCD155 and an agonistic antibody that enhances release of IFN-γ (Fig. 8A), and (iv) increased secretion of a set of cytokines involved in antiviral immune responses and IL-17 signaling upon CD96 ligation (Fig. 8, C to F). To extent our immunophenotyping and functional analysis, we combined the approach with genetic manipulation of CD4^+^ T cells and depleted CD96 via CRISPR-Cas9. The data indicated that basal expression of CD96 on CD4^+^ T cells is associated with coregulation of cell adhesion and migration (Fig. 6A). In vivo, we confirmed that CD96-expressing T cells in the embryonic thymus exhibited altered migratory behavior. Even not impaired in speed, CD96-expressing T cells exerted altered straightness, demonstrating a role of CD96 in regulating T cell migration. All these data are in line with CD96 interaction to the Nectin family receptors, which play an essential role in cellular adhesion processes (*53*, *73*). Last, we show that HIV-1–infected primary CD4^+^ T cells are less prone to adhere to the CD96 ligand CD155 in a Vpu- and Nef-dependent manner (Fig. 7F) and that HIV-1–infected cells are less responsive to CD96-induced IFN-γ secretion when they express Nef and Vpu, corresponding to reduced cell surface levels of CD96 (Fig. 8B).

There is additional evidence supporting an important role of CD96 in vivo in the context of HIV-1 pathogenesis and disease progression. Reduced CD96 expression in the overall population of CD8^+^ T cells was reported in PLWH, and this loss of CD96 expression was associated with poor response to HIV-1 antigens and a poor prognosis (*74*, *75*). How this reduced CD96 expression on CD8^+^ T cells, which are not productively infected by HIV-1, is achieved is currently unclear. One possibility is an immunological feedback loop with infected CD4^+^ T cells or the potential secretion of accessory viral proteins in exosomes that could act in trans on noninfected bystander cells. The latter was reported for Nef, which is supposed to be present in exosomes secreted from infected cells (*76*, *77*). Regardless, in conjunction with the data presented herein, CD96 emerges as previously unknown player in HIV-1 immunopathogenesis and beyond. We propose a previously unidentified role for CD96 in CD4^+^ T cells: to regulate and activate antiviral T_H_1/T_H_17-driven immune responses and signaling, which is why HIV-1 evolved potent countermeasures and efficiently removes CD96 from the surface of infected cells.

## MATERIALS AND METHODS

Antibodies and recombinant proteins used, as well as the specific application, the working dilutions, and the source, are detailed in Table 1.

**Table 1.** Antibodies and recombinant proteins used.

Name	Application	Dilution/working concentration	Company	Catalog number
Goat anti-rabbit (IRDye 800CW)	WB	1:15,000 in TBS-T	LI-COR	926-32211
Goat anti-mouse (IRDye 680RD)	WB	1:15,000 in TBS-T	LI-COR	926-68070
Goat anti-mouse (IRDye 800CW)	WB	1:15,000 in TBS-T	LI-COR	926-32210
Goat anti-human (IRDye 800CW)	WB	1:15,000 in TBS-T	LI-COR	925-32232
Donkey anti-goat (IRDye 800CW)	WB	1:15,000 in TBS-T	LI-COR	926-32214
Donkey anti-goat (IRDye 680RD)	WB	1:15,000 in TBS-T	LI-COR	926-68074
Anti-hCD96 (sheep)	WB	1 μg/ml in 5% BSA TBS-T	R&D Systems	AF6199
Anti-GFP/YFP/CFP (rabbit)	WB	1:1000 in 5% milk TBS-T	BioVision	3999-100
Anti-actin (mouse)	WB	1:1000 in 5% milk TBS-T	Sigma-Aldrich	A3853
Anti-streptavidin (IRDye 800CW)	Cytokine array	1:1000 in blocking buffer	LI-COR	926-32230
PE anti-hCD96	Flow cytometry	1:50–1:20 in 1% FBS in PBS	BioLegend	338406
PE/Cy7 anti-hCD96	Flow cytometry	1:50–1:20 in 1% FBS in PBS	BioLegend	338416
BV421 anti-hCD96	Flow cytometry	1:50–1:20 in 1% FBS in PBS	BioLegend	338418
PE anti-human CD155	Flow cytometry	1:50 in 1% FBS in PBS	Miltenyi Biotec	130-105-905
PE/Cy7 anti-human CD226	Flow cytometry	1:50 in 1% FBS in PBS	BioLegend	338316
PE-Vio770 anti-human NTB-A	Flow cytometry	1:50 in 1% FBS in PBS	Miltenyi Biotec	130-105-596
KC57 (p24) HIV-1 core antigen–RD1	Flow cytometry FACS	1:50 in 1% FBS in PBS	Beckman Coulter	6604667
KC57 (p24) HIV-1 core antigen–fluorescein isothiocyanate	Flow cytometry	1:50 in 1% FBS in PBS	Beckman Coulter	6604665
Purified anti-human CD3 (clone HIT3a)	Stimulation	0.5–2 μg/ml	BioLegend	300302
Purified anti-human CD28 (clone CD28.2)	Costimulation	0.5–1 μg/ml	BioLegend	302902
Purified anti-hCD96 (TACTILE) antibody (clone NK92.39)	Costimulation PBMCs for IFN-γ release	5 μg/ml	BioLegend	338402
Human CD155 (PVR) Fc chimera recombinant protein	Costimulation	5 μg/ml	Thermo Fisher Scientific	A42502
Recombinant human CD155-Fc chimera (carrier-free)	Adhesion assay	5 μg/ml	BioLegend	785204
Recombinant human Nectin-1-Fc chimera (carrier-free)	Adhesion assay	5 μg/ml	BioLegend	578904
Recombinant anti-hCD96 REA (clone REA195)	Costimulation CD4^+^ T cells for cytokine array and adhesion assay	2.5 μg/ml	Miltenyi Biotec	130-095-212
REA control human IgG1	Costimulation CD4^+^ T cells for cytokine array and adhesion assay	2.5 μg/ml	Miltenyi Biotec	130-129-977
PE anti-human CD4	Flow cytometry	1:50 in 1% FBS in PBS	BioLegend	300508
PE anti-human CD317	Flow cytometry	1:50 in 1% FBS in PBS	BioLegend	348406
PE anti human HLA-ABC,	Flow cytometry	1:50 in 1% FBS in PBS	BioLegend	311406
Anti–HIV-1 p24 (mouse; clone MAK183)	ELISA (coating antibody)	1:1000 in PBS	Exbio	CM-IgG
Anti–HIV-1 p24 (rabbit)	ELISA (first antibody)	1:650 in 10% FBS PBS-T	Eurogentec	-
Anti–rabbit peroxidase	ELISA (second antibody)	1:2000 in 10% FBS PBS-T	Dianova	111-035-008
T-Track CMV RUO Kit	ELISpot	According to the manufacturer’s protocol	Mikrogen Diagnostics	12201002
LEGENDScreen PE Kit	Flow cytometry	According to the manufacturer’s protocol	BioLegend	700001
IFN-γ secretion assay detection kit PE	Flow cytometry	According to the manufacturer’s protocol	Miltenyi Biotec	130-054-202
IL-2 secretion assay (hum) detection kit PE	Flow cytometry	According to the manufacturer’s protocol	Miltenyi Biotec	130-090-487
IL-4 secretion assay detection kit PE	Flow cytometry	According to the manufacturer’s protocol	Miltenyi Biotec	130-054-102
IL-17 secretion assay detection kit PE	Flow cytometry	According to the manufacturer’s protocol	Miltenyi Biotec	130-094-537

### Proviral constructs and plasmids

The HIV-1 pBR-NL4-3 IRES-eCFP proviral construct has been described previously (*78*). Similarly, an mTagBFP-expressing version and *nef*/*vpu*-deficient variants were generated by exchanging the IRES-eGFP cassette of previously described pBR–NL4-3–IRES–eGFP constructs containing single or combined inactivating mutations in *nef* and *vpu* (*5*) with IRES-mTagBFP using Mlu I and Xba I restriction sites. pBR–NL4-3–IRES–eGFP constructs containing deletions in *vpr* have been described (*79*). *Nef*-deficient pBR–NL4-3–V3 92th014.12_iGag constructs expressing various Vpu mutants were generated through cloning of *vpu* mutant sequences from pCG-IRES-GFP plasmids into the pBR–NL4-3–V3 92th014.12_nef-VpuStop-iGag-GFP vector. The pBR–NL4-3–nef–VpuStop–iGag–GFP vector was initially constructed by isolating the *vpu*–Nco I/Sac II cassette from the pBR–NL4-3 Nco I/Sac II NL4-3–*vpu* Stop proviral plasmid (*7*) using Age I and Nhe I restriction enzymes, followed by ligation of the cassette into the pBR–NL4-3–V3 92th014.12_nef-_iGag-GFP (*80*). The *vpu* mutants of interest were subsequently amplified by polymerase chain reaction, incorporating Nco I and Sac II restriction sites at the termini of the respective *vpu* genes. Final cloning was performed by restriction digestion with Nco I and Sac II to facilitate insertion of the *vpu* mutants into the *nef*-deficient pBR–NL4-3–iGag–GFP backbone. Plasmids encoding different primary alleles and mutants of Nef and Vpu (Fig. 3, A to C) along with GFP from a bicistronic mRNA (pCG-IRES-GFP) have been previously described (*7*, *9*, *10*, *40*, *81*–*83*), as well as the WITO Vpu expression constructs (*79*, *84*) and CD96v1 and CD96v2 expression constructs (*20*). pmScarlet (#85042) and Lamp1-mScarlet (#98827) were obtained from Addgene, while pmScarlet–Golgi-7 was generated by subcloning the B4GALT1-coding region from mEmerald–Golgi-7 (Addgene #54108) into pmScarlet using Nhe I/Bsh TI restriction sites. Similarly, pmScarlet-TGN was generated by subcloning the TGOLN2-coding region from mEmerald–TGNP-N-10 (Addgene #54279) into pmScarlet using the same restriction sites. Lck:CD96-GFP was generated by subcloning of the CD96-coding region from the CD96v2 expression construct into lck:GFP using Nhe I/Bsh TI restriction sites. All newly generated plasmids were verified by sequencing to control for proper insertion and coding sequence integrity.

### Cell culture, transfection, electroporation, and infection

293T or HeLa cells were cultured in Dulbecco’s modified Eagle’s medium (DMEM; Gibco) with 10% fetal bovine serum (FBS; Gibco), supplemented with 1% penicillin and streptomycin (P/S; Life Technologies) and 1% l-glutamine (Merck Millipore). Jurkat-TAG cells and stably transduced Jurkat-TAG-CD96v2 cells were maintained between 2 × 10^5^ and 2 × 10^6^ cells/ml in RPMI 1640 (Gibco) with 10% FBS (Gibco), 1% P/S (Life Technologies), and 1% l-glutamine (Merck Millipore), with Jurkat-TAG-CD96v2 supplemented with puromycin (1 μg/ml; Sigma-Aldrich). All cells were grown at 37°C and 5% CO_2_. Primary CD4^+^ T cells were derived from buffy coats obtained from the Transfusion Medicine Tübingen, with blood donors providing informed consent for the use of blood-derived products in research. Data on age, gender, or ethnicity were not collected, and all procedures adhered to the ethical guidelines approved by the ethics committee of the University Hospital Tübingen (Institutional Review Board nos. 507/2017BO1 and 860/2923BO1). CD4^+^ T cells were isolated by negative selection using the RosetteSep Human CD4^+^ T Cell Enrichment Cocktail (STEMCELL Technologies) according to the manufacturer’s protocol, reaching a purity of ≥98% confirmed by flow cytometry. Primary CD4^+^ T cells were cultured in RPMI 1640 (Gibco) with 10% FBS (Gibco), supplemented with 1% P/S (Life Technologies), 1% l-glutamine (Merck Millipore), and IL-2 (10 ng/ml; STEMCELL Technologies). Stimulation of the primary CD4^+^ T cells was performed with PHA (1 μg/ml; Thermo Fisher Scientific) for 3 days before infection, unless indicated otherwise. For restimulation, primary PHA-activated CD4^+^ T cells were treated with IL-2 (10 ng/ml; STEMCELL Technologies), anti-human CD3 (2 μg/ml; clone HIT3a, BioLegend), and anti-human CD28 (1 μg/ml; clone CD28.2, BioLegend) for up to 48 hours. To inhibit lysosomal or proteasomal degradation, PHA-activated CD4^+^ T cells were treated with 100 nM BafA1 (AdipoGen Life Sciences), 10 μM LC (Sigma-Aldrich), or dimethyl sulfoxide (DMSO; Sigma-Aldrich), respectively. Autologous PBMCs were extracted from buffy coat. Therefore, buffy coat was diluted 1:1 with phosphate-buffered saline (PBS) and centrifuged at 2200 rpm for 45 min over a Ficoll layer. PBMCs were isolated from the interphase, washed with PBS, resuspended in RPMI 1640 (Gibco) with 10% FBS (Gibco), supplemented with 1% P/S (Life Technologies), 1% l-glutamine (Merck Millipore), and IL-2 (10 ng/ml; STEMCELL Technologies), and kept in culture for up to 3 days. For cytotoxicity experiments, PBMCs were labeled using the PKH67 Green Fluorescent Cell Linker Kit (Sigma-Aldrich) according to the manufacturer’s protocol. Briefly, cells were stained with 2 μM PKH67 for 5 min, and staining was subsequently halted by adding FBS (Gibco), followed by two washes with RPMI 1640 containing 10% FBS (Gibco) and 1% P/S (Life Technologies).

Adherent cells were transiently transfected using either calcium phosphate or Lipofectamine 2000 (Thermo Fisher Scientific). One day before transfection, cells were counted and seeded. For calcium phosphate–based transfection of a 12-well plate, 2.5 μg of plasmid DNA was used. The plasmid DNA and 250 mM CaCl_2_ were mixed and filled up with double-distilled H_2_O. The DNA-CaCl_2_ solution was added dropwise to 2× HBS, vortexed, and spun down. After incubation for 15 min on ice, the mixture was added dropwise onto the cells, and transfection was stopped by replacing the medium at 5 to 16 hours posttransfection. For transfection using Lipofectamine 2000 (Thermo Fisher Scientific), 5 μg of DNA was diluted in Opti-MEM (Gibco) in total volume of 100 μl. The Lipofectamine solution consisting of 8 μl of Lipofectamine 2000 (Thermo Fisher Scientific) and 92 μl of Opti-MEM (Gibco) was added to the DNA solution and incubated for 15 min at room temperature (RT). The transfection mix was added dropwise onto the cells, and transfection was stopped by replacing the medium at 4 hours posttransfection.

Jurkat-TAG cells were electroporated using the Invitrogen Neon Microelectroporator according to the manufacturer’s protocol. Briefly, 200 000 cells per electroporation were washed with PBS, resuspended in 10 μl of Neon Buffer R, and electroporated with 2 μg of plasmid DNA at 1325 or 1600 V for 10 ms with three pulses. Following electroporation, cells were cultured in 12-well plates with 1 ml of prewarmed RPMI 1640 (Gibco) with 10% FBS (Gibco) for 24 to 48 hours.

HIV-1 virus stocks were produced by standard phosphate calcium transfection of 293T cells as described previously (*78*, *79*), and virus stocks were harvested 40 hours posttransfection. For infection, prestimulated CD4^+^ T cells [PHA (1 μg/ml) for 3 days] were incubated with the according virus stock supplemented with IL-2 (10 ng/ml) up to 72 hours.

### Cas9 RNP–mediated gene editing of primary human CD4^+^ T cells

For gene editing, Cas9 RNPs were assembled and electroporated into human CD4^+^ T cells using the Invitrogen Neon Microelectroporator. RNAs were ordered at Horizon Discovery LTD and are detailed in Table 2.

**Table 2.** RNAs used for CRISPR-Cas9 KO.

Name	Function	Target sequence	Genomic location and protospacer adjacent motif	Catalog number
Edit-R CRISPR-Cas9 synthetic tracrRNA	Transactivating CRISPR RNA	–	–	U-002005
Edit-R hCD96 (10225) crRNA	CD96 targeting CRISPR RNA #2	ACTTACCACCGACCATGCAT	hg38|-chr3:111567631–111567653 AAG	CM-020045-02
Edit-R hCD96 (10225) crRNA	CD96 targeting CRISPR RNA #4	TACCGTGTGTCTGAATGAGA	hg38|-chr3:111545383–111545405 AGG	CM-020045-04-0002

The RNAs were resuspended in 10 mM tris-HCl (pH 7.4) to the desired final concentration and incubated for 30 min at RT. The RNAs were stored in small aliquots at −80°C.First, tracrRNA (40 μM) and crRNA (40 μM) were mixed in a 1:1 ratio, and the guide RNA (gRNA) complex was assembled for 30 min at 37°C. Cas9 protein (40 μM) was added in equal volumes to the gRNA, and Cas9 RNPs were generated by incubation 15 min at 37°C. For each electroporation, 250,000 PHA-stimulated CD4^+^ T cells were washed with PBS, resuspended in 9 μl of Neon Buffer T, and combined with 1 to 2 μl of Cas9 RNPs (20 μM). Electroporation was carried out at 1600 V for 10 ms with three pulses using the Invitrogen Neon Microelectroporator. Cells were subsequently cultured in prewarmed RPMI 1640 (Gibco) with 10% FBS (Gibco). KO efficiency was analyzed by flow cytometry 2 to 3 days postelectroporation.

### RNA-seq of the SupT1 CD4^+^ T cell line

SupT1 CD4^+^ T cells were lentivirally transduced with the lentiCrispRv2 system, either with the control vector or with the lentiviral vector encoding a CD96-targeting single gRNA. Target sequence for the Lenti-CrispRv2 CD96 KO is CCAGTCCAAATCTTCGATGA. Before harvesting and RNA isolation, cells were controlled for viability and CD96 expression levels via flow cytometry. RNA was isolated via the RNeasy Kit (QIAGEN) according to the manufacturer’s protocol including the QIA Shredder column and deoxyribonuclease digestion steps. The RNA drying step was prolonged to 5-min incubation. After RNA isolation, an aliquot was taken for RNA quantification via the NanoDrop spectrophotometer (Thermo Fisher Scientific) and immediately frozen at −20°C. RNA quality assessment and subsequent sequencing of the RNA, as well as bioinformatic processing of the results, were done via the core facility of the Quantitative Biology Center (QBiC) Tübingen. All genes analyzed are accessible via dataset S3.

### HIV-1 p24 capsid antigen ELISA

To quantify HIV-1 p24 capsid antigen, an in-house developed p24-ELISA was used (protocol provided by C. Goffinet). Therefore, virus stocks or cell supernatants were lysed with Triton X-100 in a final concentration of 1% at 37°C for 2 hours or at 4°C overnight (O/N). Ninety-six–well plate (Nunc Immuno Plate, Maxi Sorb Surface) was coated with anti–HIV-1 p24 (mouse; clone MAK183, ExBio) O/N in a humidified chamber at RT and washed with PBS-T (0.05% Tween 20), followed by blocking with 10% FBS in PBS for 2 hours at 37°C. HIV-1 p24 capsid protein (ab43037, Abcam) was used in a 1:2 dilution series (50 ng/ml to 97 pg/ml) to generate a standard curve. Samples were diluted in four different concentrations with 0.05% Triton X-100 in PBS-T. Standard and samples were incubated O/N in a humidified chamber at RT. Unbound material was removed by several washing steps with PBS-T. Primary anti–HIV-1 p24 antibody (rabbit, Eurogentec) and secondary anti–rabbit peroxidase antibody (111-035-008, Dianova) were incubated each for 1 hour at 37°C. SureBlue tetramethylbenzidine peroxidase substrate (SeraCare) was added, and reaction was stopped by adding 0.5 M H_2_SO_4_ (AppliChem). Absorbance was measured at 450 and 650 nm with Cytation multiplate reader (BioTek). Absolute p24 values (in nanograms per milliliter) were calculated from the standard curve.

### Cytokine secretion assay

PHA-prestimulated primary CD4^+^ T cells were assessed for cytokine secretion using the cytokine secretion assay to detect human IL-2, IL-4, IL-17, and IFN-γ, following the manufacturer’s instructions. Briefly, cells were washed with 1× PBS supplemented with 0.5% bovine serum albumin (BSA) (Roth) and 2 mM EDTA (Sigma-Aldrich) and then incubated on ice for 5 min with capture reagents specific for human IL-2, IL-4, IL-17, and IFN-γ (cytokine secretion assay kits, Miltenyi Biotec, Auburn, CA). Cells were subsequently resuspended in warm assay medium at a concentration of 1 million cells/ml and rotated at 37°C for the durations specified in each experiment to facilitate cytokine secretion and binding to the capture reagents. Following cytokine capture, cells were washed with 1× PBS containing 0.5% BSA and 2 mM EDTA. Cells were stained with antibodies targeting specific surface markers and cytokines. After a 60-min incubation on ice, cells were washed again, and surface staining for CD96 was performed. Dead cells were excluded from flow analysis by adding 7-AAD (Miltenyi Biotec, Auburn, CA). To assess IFN-γ secretion of infected cells upon CD96 costimulation, resting CD4^+^ T cells were infected with HIV-1 NL4-3 WT IRES-eGFP or HIV-1 NL4-3 N^−^/U^−^ IRES-eGFP and stimulated with anti-CD3/anti-CD28 (1 μg/ml each; clones HIT3a and CD28.2, BioLegend) at 6 hours postinfection. Two days postinfection, cells were restimulated with anti-CD3/anti-CD28 (0.5 μg/ml each; clones HIT3a and CD28.2, BioLegend) alone or in combination with anti-CD96 (2.5 μg/ml; clone REA195, Miltenyi Biotec) or with an isotype control antibody (2.5 μg/ml; REA control human IgG1, Miltenyi Biotec). Three days poststimulation, cytokine secretion was measured. CD3, CD96, and isotype control antibodies were coated onto culture plates in PBS for 2 hours at 37°C or O/N at 4°C before seeding cells.

### Cytokine array

Anti-CD3/anti-CD28 (0.5 μg/ml each)– and mock-, anti-CD96 (2.5 μg/ml)–, or isotype control antibody (2.5 μg/ml)–costimulated primary CD4^+^ T cells were assessed for cytokine secretion using the human cytokine array C5 (RayBiotec), allowing for the detection of 80 different cytokines. Supernatants of (co)stimulated cells were harvested 3 days poststimulation and were either stored at −20°C or were directly used for cytokine detection according to the manufacturer’s protocol, unless stated otherwise. If stored at −20°C, then samples were centrifuged at high speed for 3 min as intended by the manufacturer. Samples were incubated O/N at 4°C with slow agitation to increase signal intensity. All following incubations were performed for 2 hours at RT. A streptavidin-CW800 IRDye from LI-COR diluted in the kit’s blocking buffer was used to allow detection with the Odyssey Fc Imaging System (LI-COR). CD3, CD96, and isotype control antibodies were coated onto culture plates in PBS for 2 hours at 37°C or O/N at 4°C before seeding cells.

### Flow cytometry analysis and screening

All working steps were performed with precooled reagents, on ice, and in centrifuges cooled to 4°C. The cells were washed with PBS, harvested in FACS buffer, and centrifuged at 1500 rpm for 5 min. Cells were incubated with the appropriate antibody (see antibody table below) for 30 min on ice in the dark and washed three times with FACS buffer afterward. Cells were resuspended in FACS buffer for immediate FACS analysis or incubated in 2% paraformaldehyde (PFA) for fixation and stored at 4°C. FACS measurements were performed using the MACSQuant VYB Analyzer (Miltenyi Biotec, Bergisch Gladbach, Germany) equipped with 405-, 488-, and 561-nm lasers.

Surface staining of HIV-1–infected human CD4^+^ T cells was performed using the LEGENDScreen Human Cell Screening (PE) Kit (BioLegend, San Diego, CA) according to the manufacturer’s protocol. The kit contains 332 PE-conjugated mAbs against human cell surface markers, as well as 10 mouse, rat, and hamster Ig isotype controls. Acquisition was performed with an LSRFortessa (BD Biosciences) and analyzed with BD FACS DIVA 7.0 (BD Biosciences) or with the MACSQuant VYB (Miltenyi Biotec, Bergisch Gladbach, Germany) and with FlowLogic software (Miltenyi Biotec). For determination of background signal intensity, the threshold was set to the MFI of the highest measured Ig isotype control for every donor.

### CD4^+^ T cell adhesion assay

Primary CD4^+^ T cells were prestimulated with anti-CD3 (2 μg/ml; clone HIT3a, BioLegend) and anti-CD28 (1 μg/ml; clone CD28.2, BioLegend) for 3 days. Cells were then either mock infected or infected with NL4-3–IRES–eGFP or NL4-3 N^−^U^−^ IRES-eGFP. At 48 hours postinfection, cells were washed with PBS, counted, and resuspended in PBS at a concentration of 2 × 10^5^ cells per 100 μl. In parallel, low-binding 96-well plates (Falcon) were either left uncoated or coated with specific CD96 ligands. Coating was performed by incubating wells with 70 μl of PBS containing either recombinant human Nectin-1 (5 μg/ml; BioLegend), CD155 (5 μg/ml; BioLegend), anti-CD96 antibody (2.5 μg/ml; clone REA195, Miltenyi Biotec), or an isotype control antibody (2.5 μg/ml; REA control human IgG1, Miltenyi Biotec) for 2 hours at 37°C. After incubation, wells were washed twice with PBS and subsequently blocked with 0.5% BSA in PBS for 1 hour at 37°C. Plates were again washed twice with PBS before cell seeding. Cells were added to the wells at 2 × 10^5^ cells per 100 μl in PBS and incubated at 37°C for 45 min to allow adhesion. Following incubation, 100 μl of PBS was gently added to each well, and nonadherent cells were carefully transferred to a fresh 96-well U-bottom plate without disturbing adherent cells. Remaining adherent cells were thoroughly resuspended in PBS supplemented with 1% FBS and transferred to a separate 96-well U-bottom plate. Complete removal of cells was confirmed microscopically. All collected cells were fixed in 2% PFA for 30 min at RT and analyzed by flow cytometry. Adhesion was calculated as the percentage of adherent cells relative to the total number of cells (adherent + nonadherent).

### Western blotting and coimmunoprecipitation

For Western blotting, cell lysates were prepared by washing cells with 1× PBS, lysing in radioimmunoprecipitation assay buffer (10 mM tris-HCl, 1 mM EDTA, 0.5 mM EGTA, 140 mM NaCl, 0.1% Na-deoxychalate, 0.1% SDS, and 1% Triton X-100) for 30 min on ice. Under reducing conditions, 6× SDS was added to the lysates, denatured by boiling at 95°C for 10 min, and subjected to SDS-PAGE. As indicated, lysates were additionally treated with PNGase F (New England Biolabs) to remove any N-linked glycans, following the manufacturer’s instructions. Proteins were transferred to a nitrocellulose membrane using the PROTEAN wet blotting system (Bio-Rad). Membranes were blocked in 5% nonfat dry milk (AppliChem) in tris-buffered saline (TBS). The respective primary antibody was in 5% nonfat dry milk (AppliChem) in TBS-T, and membranes were incubated at 4°C O/N. After primary antibody incubation, membranes were washed in TBS-T (10 min, three times), followed by incubation with the respective species-specific secondary antibody diluted in 5% nonfat dry milk (AppliChem) in TBS-T. Secondary antibodies conjugated to Alexa Fluor 680/800 fluorescent dyes were used for detection with the Odyssey Fc Imaging System (by LI-COR). Bands of interest were quantified using the LI-COR system and controlled for variability by normalizing to a housekeeping gene, e.g., actin.

For coimmunoprecipitation, 293T cells transfected with 4 μg of DNA of CD96v1-BioID2-HA and 4 μg of DNA of Nef or Vpu (pCG-AU1 vectors) were lysed in 500 μl of immunoprecipitation lysis buffer [50 mM tris, 0.5% Brij 98, 150 mM NaCl, 5 mM Na_3_VO_4_, 5 mM NaP_2_O_7_, 5 mM NaF, and protease inhibitor cocktail (Roche)]. Lysates were precleared with empty Sepharose beads (Sigma-Aldrich) for 1 hour at 4°C. Immunoprecipitation was performed at 4°C for 1 hour using 2 μg of anti-HA antibody. Thereafter, lysates were incubated with protein G Sepharose beads (Sigma-Aldrich) for 1 hour at 4°C and extensively washed in lysis buffer, and 3× SDS loading was added to the immunoprecipitations. Proteins were denatured by boiling for 10 min at 95°C, centrifuged, and subjected to Western blotting for protein identification.

### Fluorescence confocal microscopy

HeLa cells were seeded on coverslips in 12-well plates with 1 ml of complete DMEM. At 24 hours postinfection and 48 hours posttransfection, HeLa cells were fixed with 2% PFA for 10 min at RT. Following fixation, cells were washed with 1× PBS and mounted on microscope slides with VECTASHIELD. Confocal microscopy was conducted using the DeltaVision OMX SR system (GE Healthcare), and image analysis and quantification were performed using Imaris software (Bitplane AG, v9.3.0).

### In vivo imaging of CD96-expressing T cells

The Cab inbred medaka fish (*Oryzias latipes*) were maintained in accordance with German animal welfare regulations (Tierschutzgesetz §11, Abs. 1, Nr. 1, husbandry permit nos. 35/9185.46/UniTÜ). In this study, only embryos and freshly hatched yolk-sac larvae preceding the legal onset of independent animal life were used. The transgenic lck:GFP line was described previously (*50*). To express CD96v2 in medaka T cells, we established a construct in which CD96v2 was under the control of the medaka lck promoter (*50*, *52*). The DNA construct was coinjected with I-Sce I meganuclease enzyme into blastomeres at one-cell stage embryos using the FemtoJet 4i microinjector. Only embryos that exhibited GFP expression in the thymus were used for analysis. The ZEISS LSM 710 confocal microscope was used to image the thymus region. Z-stacks (*z* = 1 μm) were acquired every 45 s over a period of 20 min, totaling approximately 30 iterations per stack. Image data analysis was conducted using the Imaris software as described previously (*50*). Briefly, cell tracking was performed in four dimensions for GFP^+^ cells (spot diameter, 3 μm; max distance, 4 μm; max gap size, 3 μm), with manual verification to include only cells within the thymus.

### ELISpot assay

ELISpots were performed via the T-Track CMV RUO Kit. PBMCs were isolated from buffy coat. The cell number was adjusted to 2 × 10^6^ cells/ml in AIM V medium (AlbuMAX supplemented, catalog no. 31035025, Thermo Fisher Scientific), and the test stripes were prepared with 50 μl of the different working solutions per well and one operator control for each test stripe only containing 150 μl of medium. Afterward, 100 μl of the prepared PBMC suspension was carefully added per well, and the stripes were incubated for 21 hours at 37°C. Besides stimulation with the CMV-specific peptide pp65 according to the manufacturer’s protocol for all conditions, CD96-specific stimulation was performed with either sCD155 (5 μg/ml), anti-CD96 (5 μg/ml, clone NK92.39, BioLegend), or a combination of both reagents in duplicates. To detect the IFN-γ–producing effector cells, the cell suspension was discarded, and the wells were washed six times with buffer WB1. Afterward, 100 μl of the primary antibody mix was added per well after diluting 5 μl of antibody in 900 μl of buffer DB. The antibody solution was incubated for 2 hours at RT. After discarding the solution, stripes were washed three times with 200 μl of WB1 and three times with WB2. For IFN-γ staining, 50 μl of staining solution were added per well and incubated for 7 min. Last, staining solution was discarded, and stripes were washed three times with tap water to stop the reaction. The stripes were then dried O/N and evaluated via AID iSpot ELISpot FluoroSpot Reader.

### Software and statistical analysis

Flow cytometric analyses were performed using FlowLogic (v8.3) unless otherwise indicated. Statistical analyses were performed using GraphPad Prism (v10.1.1) and Microsoft Excel. Figures were generated using GraphPad Prism (v10.1.1) and CorelDraw 2024 (v25.0.0.230). Additional software used included Bitplane Imaris V9.30 and V9.9.0, ImageJ software packages (Fiji), Image Studio Lite Ver 5.2, and Microsoft Office (Word, PowerPoint, and Excel). Pathway enrichment analyses were conducted using Enrichr (*85*) (https://maayanlab.cloud/Enrichr) or Metascape (*86*) (www.metascape.org).

## Supplementary Materials

### The PDF file includes:

Figs. S1 and S2 Legends for datasets S1 to S4 Legends for movies S1 and S2

### Other Supplementary Material for this manuscript includes the following:

Datasets S1 to S4
