# Supplementary material for: HIV-1 manipulates CD96 on CD4+ T cells to subvert antiviral immunity
Source: Sci Adv. 2025 Sep 5;11(36):eadx7485. doi: 10.1126/sciadv.adx7485 (PMC12412668; doi:10.1126/sciadv.adx7485)
Supplement: Supplementary file 1 — Figs. S1 and S2 Legends for datasets S1 to S4 Legends for movies S1 and S2 [file sciadv.adx7485_sm.pdf]

Science Advances
AAAS

Supplementary Materials for

HIV-1 manipulates CD96 on CD4^{+} T cells to subvert antiviral immunity

Sandra Dehn *et al.*

Corresponding author: Michael Schindler, michael.schindler@med.uni-tuebingen.de

Sci. Adv. **11**, eadx7485 (2025)
DOI: 10.1126/sciadv.adx7485

The PDF file includes:

Figs. S1 and S2
Legends for datasets S1 to S4
Legends for movies S1 and S2

Other Supplementary Material for this manuscript includes the following:

Datasets S1 to S4
Movies S1 and S2

Supplementary material

Figures S1 and S2

Titles and captions to supplementary datasets and movies

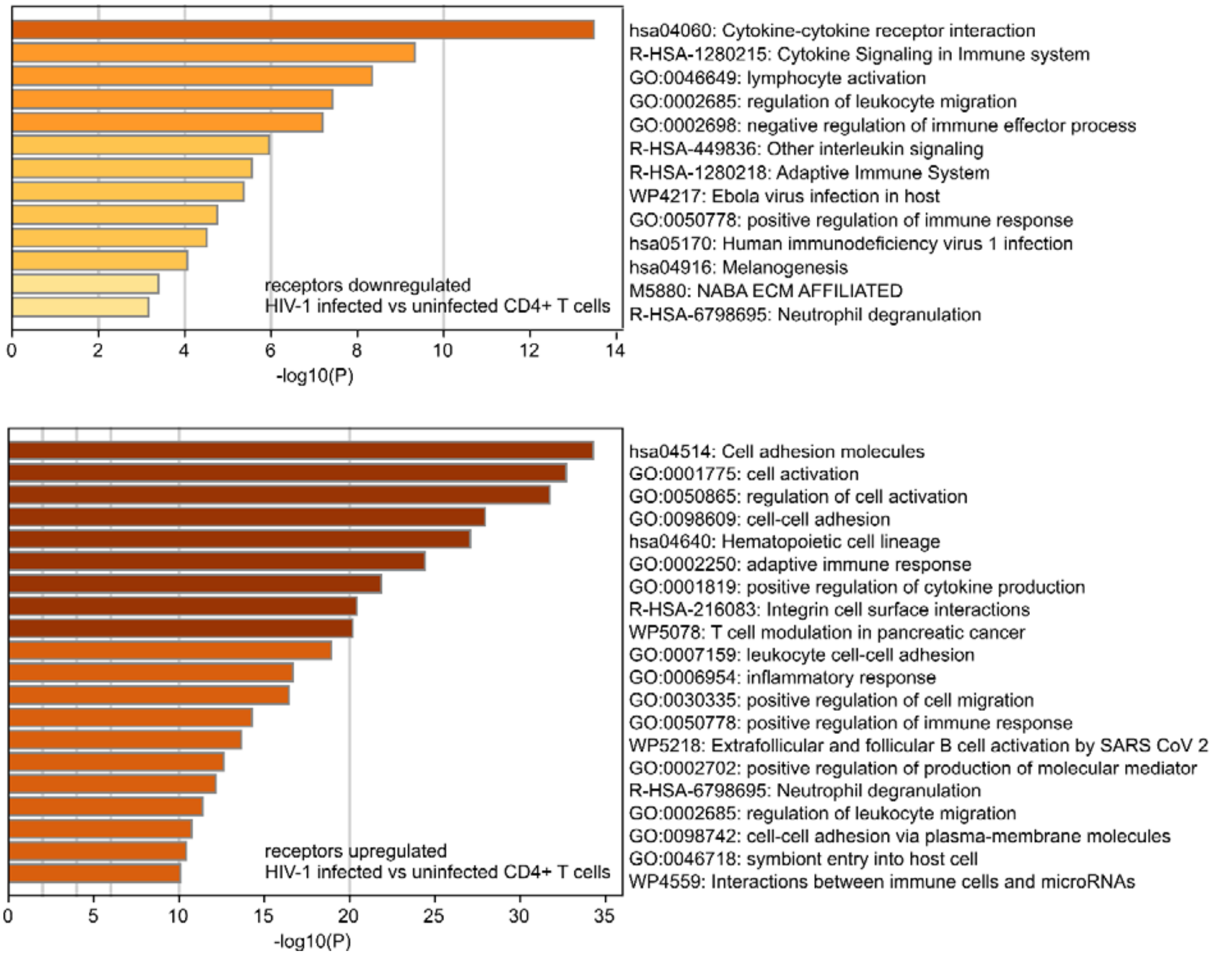

Figure S1. Meta-analysis of HIV-1 receptor modulation data. Metascape bar (www.metascape.org) showing enrichment clusters of various HIV-1-dysregulated biological processes that are connected to the 23 receptors downregulated by HIV-1 (top) and the 92 receptors being upregulated in primary CD4+ T cells. GO term analysis was done based on the significant receptor modulations detailed in Supplemental dataset 1 and shown in Figure 1.

a

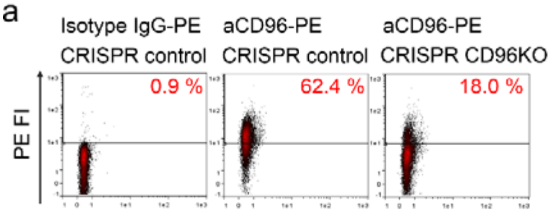

b

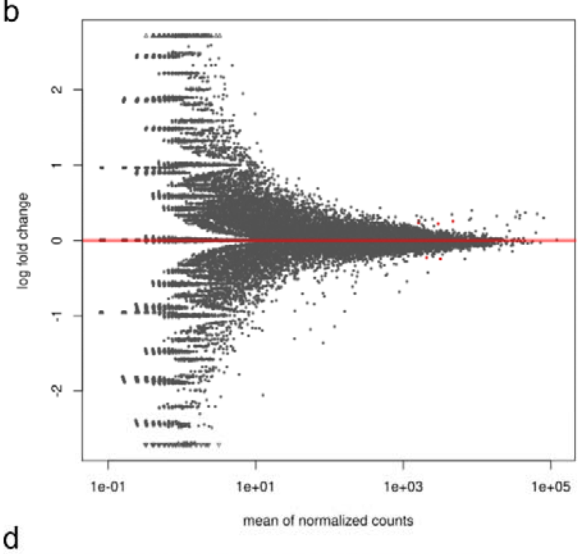

c

gene name	log2FoldChange	pvalue	padj
TAF10	-0.278666742	0.000166281	0.063519515
YBX3	-0.243535869	7.32771E-07	0.001338065
STAT1	-0.227533073	1.05084E-06	0.001338066
RFK	-0.204338037	0.000569048	0.098807451
PPP3R1	-0.201561762	0.000550636	0.098807451
IGFBP2	-0.188809935	0.000318596	0.076878349
SLBP	-0.187635028	0.000212600	0.073830276
LRRC47	-0.187197259	0.000562479	0.098807451
SQLE	-0.177885272	0.000100073	0.052307200
RAG1	-0.171068942	0.000253612	0.076878349
KPNA3	-0.153741562	0.000358032	0.080451899
VIM	-0.151041177	0.000119942	0.052307203
YWHAH	-0.149916131	0.000123236	0.052307203
HMGCR	-0.140471414	0.000322003	0.076878349
BRD9	0.186232687	0.000419281	0.088980805
KIF18B	0.190908935	0.000283046	0.076878349
CHTF18	0.211481694	0.000531367	0.098807451
ACADVL	0.217031718	2.43392E-05	0.018595126
TUBGCP6	0.233083065	1.78632E-05	0.017059315
CDK5RAP3	0.251264368	7.84610E-08	0.000299721
RHPN1	0.251605595	0.000266884	0.076878349
SPPL2B	0.261924069	5.13516E-05	0.032693850

d

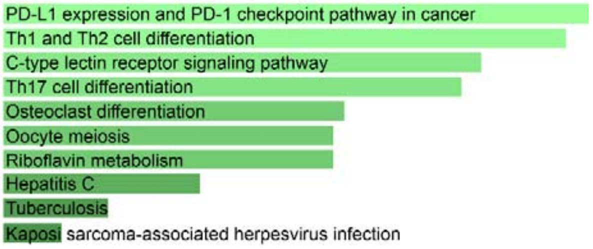

Figure S2. Differentially expressed genes in SupT1 CD4+ T cells upon knockout of CD96. (a) SupT1 CD4+ T with a non-targeting CRISPR control KO or the functional CD96KO were stained with a CD96-PE specific antibody or IgG1 isotype control and analyzed by flow cytometry, to control for efficient knockout of CD96. (b) RNA of CRISPR control and CRISPR CD96KO SupT1 cells was isolated and RNA sequencing as well as data analysis was performed via Quantitative Biology Center QBiC, Tübingen. (c) List of of differentially expressed genes upon CD96KO compared to CRISPR control (FDR adjusted p-value <0.1; three biological replicates). (d) KEGG 2021 pathway analysis ((https://maayanlab.cloud/Enrichr/) of the dereg genes from (c). The primary data is listed in Supplemental dataset 3.

Supplementary dataset 1. Primary data of flow cytometry-based cell surface receptor screening in HIV-1 infected CD4+ T cells. Raw values (mean fluorescence intensities) of the flow cytometry-based cell surface receptor screening detailed in Figure 1 for all 13 donors (tab 1). Calculation of the ratio of x-fold modulation and statistical significance via a two-tailed paired t-test (tab 2), Hit list ordered according to x-fold modulation of significant hits.

Supplementary dataset 2. Primary data of receptor enrichment on CD96 KO primary CD4+ T cells. Raw values (median fluorescence intensities) of receptors that were found to be enriched/reduced on control vs CD96 KO CD4+ T cells and calculation of log2-fold modulation from two donors (tab 1). Hit list ordered according to x-fold modulation with a threshold of 1.5 or log2 0.58-fold (tab 2). Dataset associated to Fig. 6a.

Supplementary dataset 3. Differentially expressed genes upon CD96 KO in Primary data of receptor enrichment on CD96 KO primary CD4+ T cells. Results of RNAseq analysis of SupT1 cells presented as log2-fold change in gene expression between CD96 KO and control cells as detailed in Fig. S2 (tab 1), Hit list filtered and ordered according to $P < 0.05$ (tab 2).

Supplementary dataset 4. Phenotypic characterization of $CD96^{Hi}$ vs $CD96^{lo}$ CD4+ T cells. Primary data from the flow cytometric characterization of cell surface receptor expression on $CD96^{Hi}$ vs $CD96^{lo}$ cells as detailed in Fig. 6b. Tabs 1 and 2, raw data from the two donors (median fluorescence intensities). Combined data of both donors and calculation of mean intensities and x-fold modulation (tab 3). Top hits filtered according to mean intensity > 1 and x-fold modulation > 1 (tab 4).

Supplementary video 1. Movement of CD96:GFP-expressing CD4+ T cells in living Medaka. Time-lapse imaging of CD96:GFP-expressing T cells in the thymus of Medaka as detailed in Fig. 4c.

Supplementary video 2. Movement of GFP-expressing CD4+ T cells in living Medaka. Time-lapse imaging of GFP-expressing T cells in the thymus of Medaka as detailed in Fig. 4c.
